# Reactive astrocytes transduce inflammation in a blood-brain barrier model through a TNF-STAT3 signaling axis and secretion of alpha 1-antichymotrypsin

**DOI:** 10.1038/s41467-022-34412-4

**Published:** 2022-11-02

**Authors:** Hyosung Kim, Kun Leng, Jinhee Park, Alexander G. Sorets, Suil Kim, Alena Shostak, Rebecca J. Embalabala, Kate Mlouk, Ketaki A. Katdare, Indigo V. L. Rose, Sarah M. Sturgeon, Emma H. Neal, Yan Ao, Shinong Wang, Michael V. Sofroniew, Jonathan M. Brunger, Douglas G. McMahon, Matthew S. Schrag, Martin Kampmann, Ethan S. Lippmann

**Affiliations:** 1 Department of Chemical and Biomolecular Engineering, Vanderbilt University, Nashville, TN USA; 2 Institute for Neurodegenerative Diseases, University of California, San Francisco, San Francisco, CA USA; 3 Biomedical Sciences Graduate Program, University of California, San Francisco, San Francisco, CA USA; 4 Medical Scientist Training Program, University of California, San Francisco, San Francisco, CA USA; 5 Department of Molecular Physiology and Biophysics, Vanderbilt University, Nashville, TN USA; 6 Department of Biomedical Engineering, Vanderbilt University, Nashville, TN USA; 7 Vanderbilt Brain Institute, Vanderbilt University, Nashville, TN USA; 8 Department of Neurology, Vanderbilt University Medical Center, Nashville, TN USA; 9 Department of Neurobiology, University of California, Los Angeles, Los Angeles, CA USA; 10 Department of Biological Sciences, Vanderbilt University, Nashville, TN USA; 11 Vanderbilt Memory and Alzheimer’s Center, Vanderbilt University Medical Center, Nashville, TN USA; 12 Weill Institute for Neurosciences, University of California, San Francisco, San Francisco, CA USA; 13 Department of Biochemistry and Biophysics, University of California, San Francisco, San Francisco, CA USA; 14 Chan Zuckerberg Biohub, San Francisco, CA USA

**Keywords:** Blood-brain barrier, Neuro-vascular interactions

## Abstract

Astrocytes are critical components of the neurovascular unit that support blood-brain barrier (BBB) function. Pathological transformation of astrocytes to reactive states can be protective or harmful to BBB function. Here, using a human induced pluripotent stem cell (iPSC)-derived BBB co-culture model, we show that tumor necrosis factor (TNF) transitions astrocytes to an inflammatory reactive state that causes BBB dysfunction through activation of STAT3 and increased expression of *SERPINA3*, which encodes alpha 1-antichymotrypsin (α1ACT). To contextualize these findings, we correlated astrocytic STAT3 activation to vascular inflammation in postmortem human tissue. Further, in murine brain organotypic cultures, astrocyte-specific silencing of *Serpina3n* reduced vascular inflammation after TNF challenge. Last, treatment with recombinant Serpina3n in both ex vivo explant cultures and in vivo was sufficient to induce BBB dysfunction-related molecular changes. Overall, our results define the TNF-STAT3-α1ACT signaling axis as a driver of an inflammatory reactive astrocyte signature that contributes to BBB dysfunction.

## Abstract (web summary)

Inflammation of brain endothelial cells is seen in neurodegenerative conditions and in aging. Here the authors examine the role of astrocytes in blood brain barrier function using an iPSC-derived cell co-culture model.

## Introduction

Specialized brain microvascular endothelial cells (BMECs) comprise the functional component of the blood–brain barrier (BBB), and together with supporting astrocytes, pericytes, and neurons, they collectively form the neurovascular unit (NVU). This NVU maintains the selective balance of molecules between the bloodstream and brain and its dynamic activity is essential for neural health and homeostasis^1–3^. The role of pericytes in BBB development, maintenance of healthy BBB function, and loss of BBB integrity during disease has become increasingly clear in recent years^4^. Likewise, newer studies are providing insight into how neuronal activity regulates local BBB functions^5^. In contrast, astrocytes, which were historically believed to be essential to BBB development and maintenance^6^, now have a less clear role in these activities. Seminal transplantation experiments in non-neural tissues showed that astrocytes could induce barrier properties in non-BBB endothelial cells^7,8^, and subsequent studies have shown that astrocyte-secreted factors such as sonic hedgehog and angiotensin can support BBB function^9–11^. Similarly, primary endothelial cells purified by immunopanning methods induced astrocyte precursor cells to differentiate into astrocytes via leukemia inhibitory factor (LIF) or LIF-like cytokines^12^, suggesting a close temporal and spatial correlation between endothelial and astrocyte differentiation. Yet, astrocytes arise postnatally after the initial onset of BBB properties, and there are conflicting reports as to whether astrocytes are acutely necessary to support BBB functions. Some reports show that delay or removal of astrocyte contacts with BMECs does not induce BBB disruption^13^, while a more recent study ablating astrocytes using tamoxifen-inducible cell death has indicated that astrocytes contribute to a non-redundant and crucial function in sustaining the adult BBB^14^. Hence, the role of astrocytes in BBB maintenance remains unclear.

This uncertainty extends into the role astrocytes may play in BBB disruption during disease. Recently, a subgroup of reactive astrocytes that can induce neuronal death in neurodegenerative conditions has been identified as a possible early driver of neurodegeneration. Inflammatory cytokines secreted by classically activated microglia induce the transition of normal astrocytes to this “inflammatory reactive” phenotype, which is highly enriched in both aged and neuropathologic human tissues^15–17^. While inflammation caused by microglia is implicated in neurodegeneration, reactive astrocyte-associated inflammation may also play a key role. Indeed, in mouse models of amyotrophic lateral sclerosis^18^, Parkinson’s disease^19^, and white matter injury^20^, the prevention of inflammatory astrocyte conversion is neuroprotective. However, the effects of such reactive astrocytes on the BBB are unclear. Some studies have shown that reactive astrocytes in pathological conditions adversely affect endothelial integrity via secreted proteins, such as vascular endothelial growth factor (VEGF)^21–23^ and interleukin 6 (IL-6)^22^. Astrocytes have also been shown to secrete factors that mediate leukocyte recruitment^24,25^. Likewise, diseases that exhibit increased numbers of reactive astrocytes are also typically associated with BBB disruption^26,27^. Yet, some studies have also shown that reactive astrocytes protect BBB functions after neural injury^28–30^. Hence, a detailed understanding of the molecular connections between reactive astrocyte subtypes and BBB disruption or repair remains largely unknown. Such limitations may be due in part to the heterogeneity of reactive astrocytes in vivo and the challenges associated with testing how the conversion of astrocytes to a reactive state influences BBB properties.

With the remarkable progress of human induced pluripotent stem cell (iPSC) technology, differentiation protocols now facilitate access to various cell types of NVU, which offers unprecedented opportunities to study human cell biology and cell–cell interactions in health and disease^31^. Astrocytes can be efficiently derived from iPSCs by temporal application of specific signaling factors, and newer protocols can be carried out under serum-free conditions to prevent acquisition of a reactive status^32,33^. In contrast, most protocols for generating BMEC-like cells have utilized serum-containing medium^34^, which previously prevented the study of reactive transition states in BMEC–astrocyte co-cultures. To address this issue, we recently developed serum-free strategies for differentiating iPSCs to BMEC-like cells, including the use of basal media typically employed in astrocyte cultures^35,36^. These advancements provide a highly controlled system to probe how reactive astrocyte transitions influence BBB properties in BMECs in the absence of other NVU cell types.

Here, we used the iPSC-derived BBB co-culture model to reveal that a tumor necrosis factor (TNF)-STAT3 signaling axis generates inflammatory reactive astrocytes that induce BBB dysfunction through loss of passive barrier function and upregulation of vascular cell adhesion molecule 1 (VCAM-1). We mapped astrocyte-specific pathways putatively involved in BBB disruption using RNA sequencing and various bioinformatics tools, then utilized CRISPR interference (CRISPRi) techniques to systematically silence different genes for loss-of-function studies, which identified *SERPINA3* (encoding alpha 1-antichymotrypsin (α1ACT)) as a candidate astrocyte factor causing BBB disruption. Follow-up experiments in mouse cortical explant cultures and in vivo confirmed these associations between reactive astrocytes, STAT3 activation, α1ACT, and BBB dysfunction-related molecular changes. Collectively, these results identify an inflammatory reactive astrocyte state that may be responsible for negative BBB outcomes and define α1ACT as a mediator of this process.

## Results

### GBP2^+^ reactive astrocytes are adjacent to inflamed VCAM-1^+^ blood vessels in various human neuropathological conditions

**Fig. 1:**
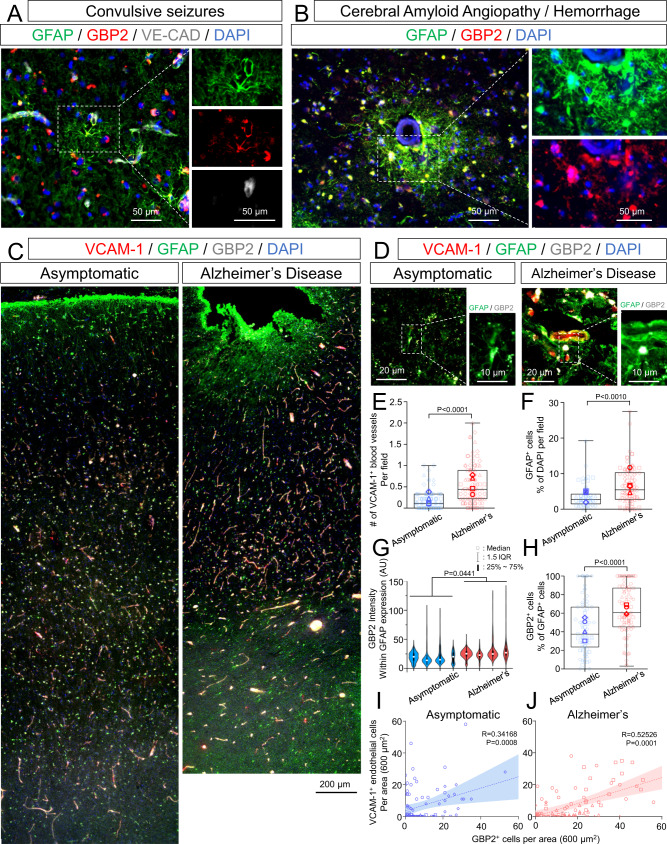
Hallmarks of GBP2^+^ inflammatory reactive astrocytes and vascular VCAM-1 expression in human brain tissue isolated from patients with neuropathological diseases. **A** Representative images of perivascular GBP2^+^/GFAP^+^ astrocytes in brain tissue from a patient with unspecified convulsive seizures. **B** Representative images of perivascular GBP2^+^/GFAP^+^ astrocytes in brain tissue from a patient with cerebral amyloid angiopathy and associated hemorrhage. **C** Representative images of VCAM-1, GFAP, and GBP2 expression across the cortex of an asymptomatic patient (left) versus a patient diagnosed with Alzheimer’s disease (right). **D** Representative cortical perivascular images of VCAM-1, GFAP, and GBP2 expression in an asymptomatic patient (left) versus a patient diagnosed with Alzheimer’s disease (right). **E**–**H** Quantification of VCAM-1^+^ vessels (**E**), GFAP^+^ astrocytes (**F**), GBP2 intensity in GFAP + astrocytes (**G**), and the percentage of GBP2^+^ astrocytes (**H**) in age-matched asymptomatic brains and Alzheimer’s disease brains (*n* = 4 biological replicates for each condition). The boxes for **E**, **F**, and **H** show the range between the 25th and 75th percentiles, the line within each box indicates the median, and the outer lines extend to 1.5 times the interquartile range (IQR) from the box. Faint datapoints indicate individual images and solid datapoints indicate the mean for each biological replicate. The violin plot (**G**) shows the same features for each biological replicate. Statistical analyses were performed with a two-sided *t* test. **I**, **J** Pearson correlation of VCAM-1^+^ cells to GBP2^+^ cells per area in age-matched asymptomatic brains (**I**) and Alzheimer’s disease brains (**J**) (*n* = 4 biological replicates for each condition). Best-fit lines with a shaded area of 95% confidence intervals from linear regression, as well as Pearson correlation coefficients and *p* values from a two-tailed test, are presented on each plot.

In advance of our in vitro studies, we sought to confirm the association between reactive astrocytes and dysfunctional BMECs in human brain tissue. Changes to the BBB and NVU, including inflamed endothelial cells and reactive astrocytes, are a common feature related to aging and neurodegenerative diseases^37,38^, but to our knowledge, the relative abundance and correlation of these changes have not been previously evaluated. In singular cases of patients exhibiting unspecified convulsive seizure disease and cerebral amyloid angiopathy with hemorrhage, we observed co-localization of GFAP and GBP2, a marker for inflammatory reactive astrocytes^17^, adjacent to blood vessels in the cerebral cortex (Fig. 1A, B). To more quantitatively investigate whether inflammatory reactive astrocytes are specifically adjacent to inflamed vessels, we probed tissue sections from an Alzheimer’s disease (AD) cohort and asymptomatic age-matched controls for GFAP, GBP2, and VCAM-1 expression. We inferred from morphology that the majority of VCAM-1 expression in the gray matter was localized to blood vessels in both aged individuals and AD patients (Fig. 1C). In addition, the expression of GBP2 was pronounced in subpopulations of GFAP^+^ astrocytes in both cohorts (Fig. 1C, D). Overall, the expression levels of vascular VCAM-1 and astrocytic GFAP and GBP2 were higher in the AD cohort compared to the age-matched asymptomatic cohort (Fig. 1E–H). Since inflammatory reactive astrocytes are known to secrete harmful cytokines that contribute to neuroinflammation, we analyzed the relationship between the expression of GBP2^+^ astrocytes and VCAM-1^+^ endothelial cells in both cohorts using a Pearson correlation test (Fig. 1I, J). As expected, a positive correlation was observed in the asymptomatic cohort (*p* = 0.0008, Pearson *r*^2^ = 0.34168) and AD cohort (*p* = 0.0001, Pearson *r*^2^ = 0.52526), suggesting interactions between dysfunctional BMECs and reactive astrocytes as a function of aging and disease.

### Soluble crosstalk between BMECs and inflammatory reactive astrocytes disrupts passive BBB function in a coculture-specific manner

**Fig. 2:**
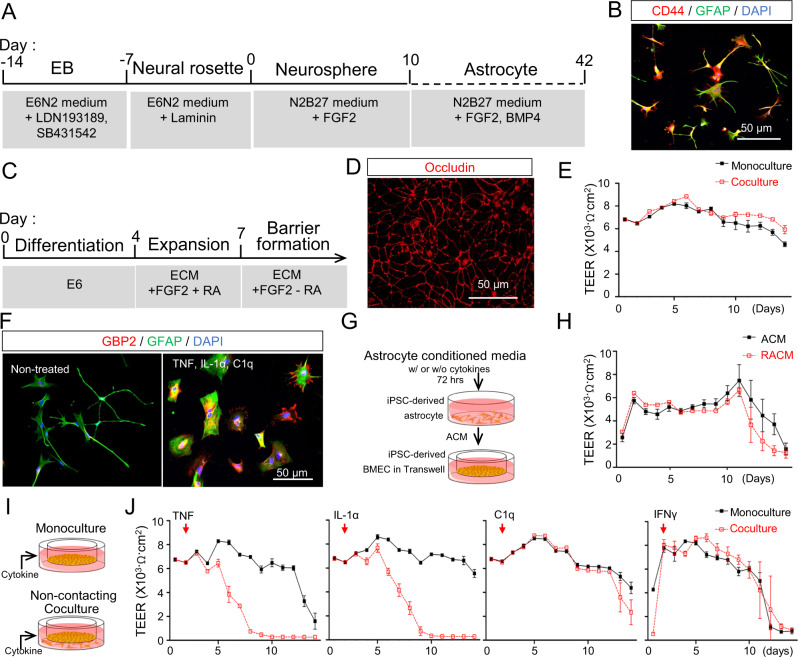
Co-culture of iPSC-derived BMEC-like cells and astrocytes is necessary for disruption of passive barrier function by inflammatory cytokines. **A** Schematic procedure for deriving astrocytes from iPSCs. **B** Representative immunofluorescent images of astrocyte markers after 40 days of differentiation. **C** Schematic procedure for deriving BMEC-like cells from iPSCs. **D** Representative immunofluorescent images of smooth and continuous tight junctions in the BMEC-like cells 8 days after differentiation. **E** Representative TEER values in BMEC-like cells in monoculture or in co-culture with astrocytes. Data are graphed as mean ± SEM from technical triplicates. Trends were confirmed across *n* = 3 biological replicates. **F** Representative immunofluorescent images of GFAP and GBP2 expression in astrocytes 48 h after treatment with an inflammatory cytokine cocktail. **G**, **H** Schematic diagram of the procedure for treating BMEC-like cells with astrocyte conditioned medium (**G**) and subsequent TEER measurements (**H**). Astrocyte conditioned medium without inflammatory cytokines is noted as “ACM” and astrocyte conditioned medium collected after cytokine treatment is referred to as reactive astrocyte conditioned medium, or “RACM.” Data are graphed as mean ± SEM from technical triplicates. Trends were confirmed across *n* = 3 biological replicates. **I**, **J** Experimental setup (**I**) and subsequent TEER measurements (**J**) after dosing BMEC-like cells with cytokines in monoculture or co-culture. Data are graphed as mean ± SEM from technical triplicates. Trends were confirmed across *n* = 3 biological replicates.

To investigate direct interactions between human BMECs and reactive astrocytes, we employed serum-free iPSC systems established in previous studies. iPSCs were differentiated to CD44^+^/GFAP^+^ astrocytes by standard neuralization, manual selection of primitive neural progenitor cells, and treatment with BMP4 and FGF2 for three weeks (Fig. 2A, B). In separate cultures, iPSCs were differentiated to BMEC-like cells under serum-free conditions, where immunostaining was used to detect continuous occludin^+^ tight junctions that afforded the cells with high transendothelial electrical resistance (TEER) that could be maintained above 1000 Ω×cm^2^ for up to 2 weeks, with or without astrocyte co-culture (Fig. 2C–E). Because secreted factors derived from quiescent astrocytes in non-contact co-culture systems support BBB properties^35^ (Fig. 2E), we first investigated if secreted factors derived from inflammatory reactive astrocytes would negatively influence TEER within the BMEC-like cells. We confirmed that treatment of the astrocytes for 48 h with the canonical inflammatory reactivity inducers interleukin 1 alpha (IL-1α), TNF, and complement subcomponent q (C1q)^17^ yielded hypertrophic ameboid shapes with GBP2 expression, indicative of transition to an inflammatory reactive state (Fig. 2F). Next, astrocytes were maintained in medium for 72 h with or without IL-1α, TNF, and C1q, and the spent medium was collected, concentrated, and fed to monocultured BMEC-like cells (Fig. 2G). Surprisingly, no significant differences in TEER were observed (Fig. 2H), suggesting active crosstalk might be necessary to transduce the damaging effects of inflammatory reactive astrocytes. To probe this possibility, we generated monocultures of BMEC-like cells and co-cultures of BMEC-like cells with astrocytes, then added single doses of various inflammatory cytokines (Fig. 2I). As expected from previous work (Fig. 2H)^39,40^, BMEC-like cells in monoculture were unresponsive to cytokine treatments. In contrast, in the co-culture system, treatment with TNF or IL-1α was sufficient to significantly reduce TEER in the BMEC-like cells. Meanwhile, C1q and interferon gamma (IFNγ) treatments did not yield BBB disruption, highlighting cytokine-specific responses (Fig. 2J). Interestingly, we observed a lag time of several days between the addition of cytokines and decreased TEER, suggesting other factors beyond the cytokines were contributing to BBB dysfunction. Hence, we next sought to analyze the changes to each cell population at later time points.

### Transcriptome profiling reveals early-stage inflammatory interactions between BMECs and astrocytes

**Fig. 3:**
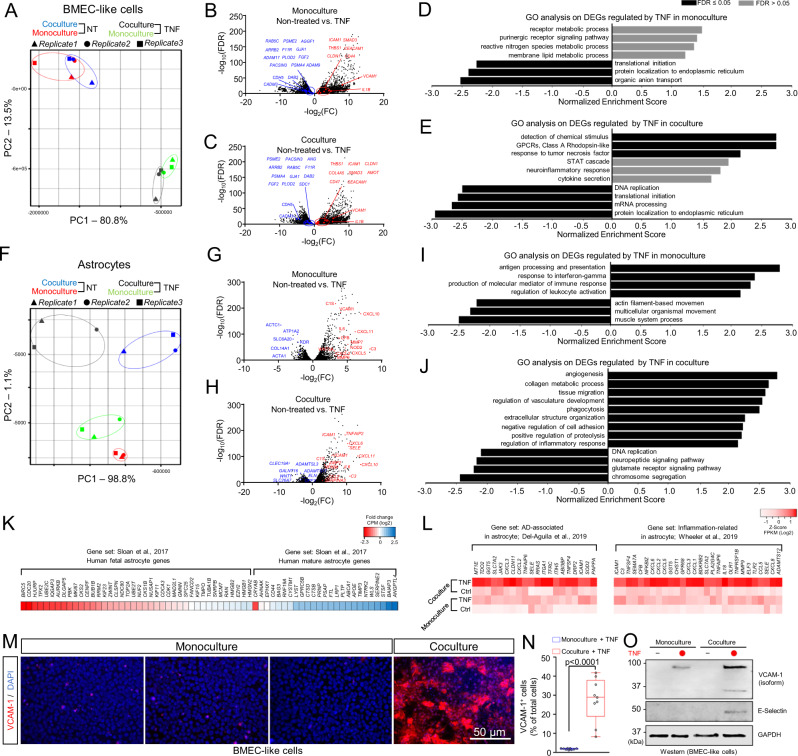
Comparison of transcriptomic signatures and inflammatory phenotypes in astrocytes and BMEC-like cells as a function of co-culture and treatment with TNF. **A** PCA clustering plot of RNA-seq data in BMEC-like cell. **B**, **C** Volcano plot of DEGs (FDR < 0.1) in BMEC-like cells in monoculture versus co-culture, with or without TNF treatment. Red and blue points highlight genes associated with endothelial cell-cell junctions, internalization and trafficking, and angiogenesis. **D**, **E** GO analyses in the BMEC-like cells in monoculture versus co-culture. **F** PCA clustering plot of RNA-seq data in astrocytes. **G**, **H** Volcano plot of DEGs (FDR < 0.1) in astrocytes in monoculture versus co-culture, with or without TNF treatment. Red and blue points highlight genes associated with inflammation, chemokines, and immune responses. **I**, **J** GO analyses in the astrocytes in monoculture versus co-culture. **K** Heat map showing fold change of gene expression of maturity-related genes in co-cultured astrocytes compared to monocultured astrocytes. The gene set is based on ref. 47. **L** Heat map showing relative expression of genes associated with AD and inflammation across all astrocyte conditions. The gene sets are based on ref. 48 (left panel) and ref. 106 (right panel). The color of the heat map represents the log2(FPKM value) for each gene. **M**, **N** Representative immunofluorescent images of VCAM-1 expression in BMEC-like cells under different conditions (**M**) and select quantification (**N**). Data were acquired at day 14 according to the timing presented in Fig. 2J. Quantification was conducted using *n* = 3 biological replicates. The box shows the range between the 25th and 75th percentiles, the line within each box indicates the median, the outer lines extend to 1.5 times the interquartile range from the box, and each data point represents an individual image (3 images quantified per biological replicate). Statistical analyses were performed with a two-sided *t* test. **O** Representative western blot showing relative VCAM-1 and E-selectin expression in BMEC-like cells in monoculture or co-culture, with or without TNF treatment. Data were acquired at day 14 according to the timing presented in Fig. 2J. GAPDH was used as a loading control. Trends were confirmed across *n* = 3 biological replicates.

To gain mechanistic insight into how reactive astrocytes contribute to BBB dysfunction in the in vitro BBB model, we constructed, mapped, and compared differences of transcriptomes and gene regulatory networks using bulk RNA sequencing (RNA-seq). First, to find an appropriate concentration of TNF for studying astrocyte/BMEC interactions, we measured TEER in response to doses of TNF ranging from 1 ng/ml to 100 ng/ml. Single TNF doses of 10 and 100 ng/ml were sufficient to cause a decrease in TEER and an increase in *VCAM1* expression in BMEC-like cells (Supplementary Fig. 1). Based on these experiments, we proceeded with a 10 ng/ml dose of TNF for transcriptional analyses, specifically isolating RNA at day 5 of culture at the time point when TEER started to decline in the co-culture condition. This time point was also used for cells in monoculture (BMEC-like cells or astrocytes) with or without TNF treatment. First, in bulk RNA-seq datasets, we checked the cellular identity of astrocytes and endothelial cells in the absence of TNF to further validate the in vitro system composed of iPSC-derived cells. In accordance with key functional properties of BBB such as elevated TEER and continuous occludin^+^ tight junctions shown in Fig. 2D, E, we could detect endothelium- and BBB-specific gene expression including *CDH5* (VE-cadherin), *TEK* (Tie2), *VWF* (von Willebrand factor), and *SLC2A1* (GLUT1) in the iPSC-derived BMEC-like cells (Supplementary Fig. 2A). Likewise, astrocytic identity was confirmed by astrocyte-specific markers including *GFAP*, *CD44*, *SLC1A2* (GLT-1), and *AQP4* (Aquaporin-4) (Supplementary Fig. 2B). To obtain a general picture of the relationship among different culture conditions, we performed unbiased dimensionality reduction with all detected transcripts using principal-component analysis (PCA) and visualized the relationship with the first two principal components. PCA on endothelial transcriptomes showed two distinct clusters defined by TNF, but not by culture condition (monoculture or co-culture with astrocytes; Fig. 3A). Volcano plots and gene ontology (GO) analyses on differentially expressed genes (DEGs) were also constructed to analyze TNF effects on monocultured versus co-cultured BMEC-like cells (Fig. 3B–E). Among the DEGs in BMEC-like cells, adhesion molecules including *VCAM1*, *CEACAM1*, and *ICAM1* were substantially induced by TNF in both monoculture and co-culture, whereas junctional adhesion molecule 1 (*F11R*) was downregulated in both monoculture and co-culture. Major genes involved in the formation and maintenance of adherence junctions, *CDH5* and *CADM3*, were also downregulated by TNF regardless of the culture system. In contrast, TNF induced the upregulation of *CD47*, an integrin-associated protein involved in age-associated deterioration in angiogenesis^41^, only in the co-culture system. Key endocytosis molecules were downregulated by TNF in both culture conditions, for instance, *PACSIN3* (important regulator of internalization of plasma membrane proteins^42^) and *RAB5C* (a small GTPases key regulator of trafficking of integrin complex in endothelial cells^43^). Exposure to TNF also caused the downregulation of the proangiogenic genes such as *AGGF1*, *FGF2*, and *ANG*^44–46^. Further, while many overlapping DEGs could be identified, we observed key differences in gene ontology. Notably, in the co-culture condition, inflammatory signaling pathways, including “response to tumor necrosis factor,” “STAT cascade,” and “neuroinflammatory response” were selectively enriched (Fig. 3D, E).

Meanwhile, the PCA on astrocytes showed both co-culture- and TNF-responsive transcriptomic signatures. The first principal component (PC1) was dominated by TNF, and the second principal component (PC2) separated the samples according to culture condition (Fig. 3F). Astrocytes treated with TNF in monoculture upregulated many inflammation-associated genes with concurrent enrichment of inflammatory signaling pathways (Fig. 3G–I), and many of these astrocytic genes were also upregulated in the TNF-treated co-culture condition (Fig. 3H–J), but some different enriched pathways associated with vascular interactions were observed, including “angiogenesis” and “regulation of vasculature development.” Based on these results, we compared the gene expression signatures in astrocytes to several published datasets. In astrocytes not treated with TNF, we observed a profound shift in gene expression towards a mature signature if astrocytes were co-cultured with BMEC-like cells (Fig. 3K)^47^. Likewise, compared to monoculture astrocytes treated with TNF, co-culture with BMEC-like cells amplified expression of inflammation- and AD-associated genes (Fig. 3L)^48^. These results suggest that iPSC-derived astrocytes acquire a more mature profile upon co-culture with BMEC-like cells, which in turn may facilitate their inflammatory responses to TNF.

The enhanced astrocytic inflammation in the co-culture condition led us to further analyze inflammatory phenotypes in the BMEC-like cells. A key molecular phenotype of endothelial cells in response to inflammation is the expression of adhesion molecules and selectins^49^. Here, we primarily focused on VCAM-1, which has recently been implicated in neurodegeneration^50^ as well as our human tissue analyses in Fig. 1. Although previous studies with human endothelial cell lines showed the prompt expression of VCAM-1 following stimulation with inflammatory cytokines^51^, a lack of adhesion molecule expression in response to inflammation has been noted as a specific shortcoming in many iPSC-derived BBB models. As described previously, in the iPSC-derived BMEC-like cells (co-cultured with astrocytes), 10 and 100 ng/ml TNF increased *VCAM1* by 2- and 4-fold, respectively, 2 weeks after co-culture in the presence of TNF (Supplementary Fig. 1B), which mirrored a decline in TEER (Fig. 2J). We therefore examined VCAM-1 protein expression on the iPSC-derived BMEC-like cells under various conditions. Immunostaining revealed minimal VCAM-1 expression in monoculture BMEC-like cells with or without TNF treatment, despite strong induction of *VCAM1* in response to TNF. Conditioned medium from TNF-treated reactive astrocytes was also unable to induce VCAM-1 expression, again indicating the importance of astrocyte-endothelial crosstalk in the inflammatory response. In contrast, in the co-culture system, TNF treatment induced widespread VCAM-1 expression (Fig. 3M, N). The effects of co-culture were confirmed by western blotting, where we also determined that E-Selectin, another key adhesion molecule, was solely induced by TNF in the co-culture condition (Fig. 3O). Overall, the results from these experiments highlight the importance of astrocyte-endothelial crosstalk for transducing inflammatory phenotypes in the in vitro BBB model, potentially through post-transcriptional mechanisms.

### TNF-mediated BBB disruption is dependent on astrocytic STAT3 signaling in the in vitro co-culture system

**Fig. 4:**
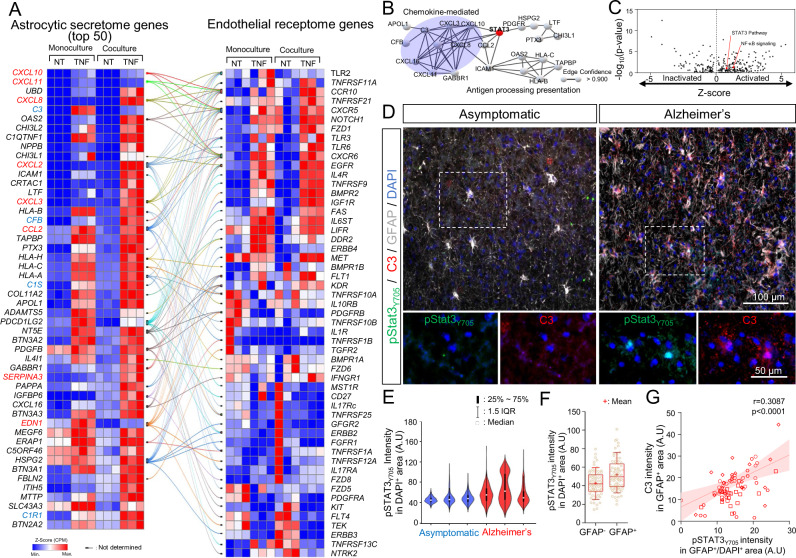
STAT3 signaling is active in reactive inflammatory astrocytes in vitro and in human tissue samples from Alzheimer’s disease patients. **A** Network analysis (confidence score >0.7) of the top 50 differentially expressed astrocytic genes encoding secreted factors, with corresponding endothelial genes encoding receptors. **B** Network analysis (confidence score >0.9) of the STAT3 interactome. **C** Ingenuity Pathway Analysis (IPA) on DEGs (FDR < 0.1) between co-cultured astrocytes with or without TNF treatment. *p* values were calculated using a right-tailed Fisher’s exact test. **D** Representative images of pSTAT3_Y705_, C3, and GFAP expression in the cortex of an asymptomatic patient versus a patient diagnosed with Alzheimer’s disease. **E** Quantification of nuclear pSTAT3_Y705_ intensity in the cortex of asymptomatic patients versus patients diagnosed with Alzheimer’s disease. Statistical analyses were performed with a one-sided *t* test. **F** Quantification of nuclear pSTAT3_Y705_ intensity in GFAP^-^ versus GFAP^+^ cells in the cortex of patients diagnosed with Alzheimer’s disease (70 cells counted per sample; *n* = 4 biological replicates). The boxes show the range between the 25th and 75th percentiles with a line and a plus sign indicating median and mean, respectively. Statistical analyses were performed with a one-sided *t* test. **G** Pearson correlation of C3 intensity in GFAP^+^ astrocytes to nuclear pSTAT3_Y705_ intensity of GFAP^+^ astrocyte in the cortex of four patients diagnosed with Alzheimer’s disease. Best-fit lines with a shaded area of 95% confidence intervals from linear regression, as well as the Pearson correlation coefficient and *p* value from a two-tailed test, are presented in the plot.

We next focused on the inflammatory signaling axis that could be responsible of TNF-mediated BBB disruption. Since BBB dysfunction in our in vitro BBB model was transduced by inflammatory reactive astrocytes in a non-contacting co-culture setup (Figs. 2J and 3M–O), we first focused on analyzing soluble factors secreted by astrocytes. To predict BBB-disruptive and immune-stimulative soluble factors provided by reactive astrocytes, we organized reactive astrocytic transcripts based on a database composed of 6,943 secreted human proteins^52^ and then computationally predicted interactions with endothelial receptome genes (Fig. 4A and Supplementary Fig. 3). Within the astrocytes, we generally observed enriched transcripts for target genes along the NF-κB/STAT3 axis that were enhanced by TNF treatment; these astrocyte genes included *CCL2*, *CXCL10*, *CXCL11*, *CXCL2*, *CXCL3*, *CXCL8*, *EDN1*, and *SERPINA3*^53–58^. We also observed enriched mapping of astrocyte-secreted factors and endothelial receptors for complement genes, including astrocytic upregulation of *C1S*, *C1RL*, *C3*, and *CFB*. Furthermore, using the Search Tool for the Retrieval of Interacting Genes/Proteins (STRING)^59^ database decoyed by STAT3, we found that 22 out of the top 50 astrocytic secretome genes were closely correlated with STAT3 (Fig. 4B). In addition, an Ingenuity Pathway Analysis conducted on DEGs predicted that NF-κB and STAT3 signaling were preferentially activated by TNF treatment in co-culture compared to monoculture (Fig. 4C).

**Fig. 5:**
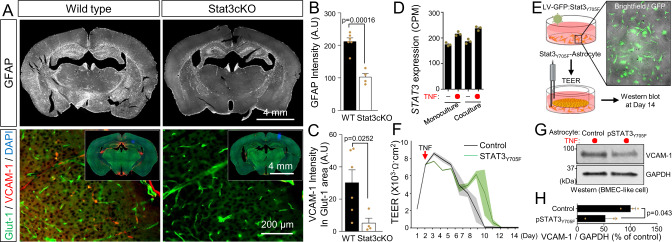
STAT3 activation in reactive inflammatory astrocytes contributes to BBB dysfunction in vitro and in vivo. **A** Representative images of GFAP, VCAM-1, and Glut-1 expression in mouse brain from a wild-type (WT) control versus astrocyte-specific Stat3 conditional knockout (Stat3cKO). **B** Bar graph showing GFAP expression in brains of WT (*n* = 6) versus Stat3cKO (*n* = 4) mice. Data are graphed as mean ± SEM. Statistical analyses were performed with a two-sided *t* test. **C** Bar graph showing VCAM-1 expression in Glut-1^+^ vessels in brains of WT (*n* = 6) versus Stat3cKO (*n* = 4) mice. Data are graphed as mean ± SEM. Statistical analyses were performed with a two-sided *t* test. **D** Bar graph showing STAT3 gene expression (CPM) in iPSC-derived astrocytes at day 5 of monoculture or co-culture, with or without TNF treatment. Data are graphed as mean ± SEM from *n* = 3 biological replicates. **E** Schematic diagram of the procedure for producing dominant-negative STAT3 iPSC-derived astrocytes for co-culture with BMEC-like cells and subsequent TEER measurements and western blot analysis. **F** TEER values in BMEC-like cells in co-culture with astrocytes after dominant negative STAT3 overexpression. Data are presented as a continuous mean ± shaded SEMs per condition from *n* = 3 technical replicates. Trends were confirmed across *n* = 3 biological replicates. **G**, **H** Representative western blot and quantification of VCAM-1 expression in BMEC-like cells. Data were acquired at day 14 according to the timing presented in Fig. 2J. GAPDH was used as a loading control. Data are graphed as mean ± SEM from *n* = 3 biological replicates. Statistical analyses were performed with a one-sided *t* test.

It is well established that the STAT3 pathway mediates astrocyte reactivity in animal models^60–62^, but fewer studies have been performed in pathological human brain samples. Typically, STAT3 activation is initiated by phosphorylation on a critical tyrosine residue (Tyr705; pSTAT3), whereby pSTAT3 is then translocated to the nucleus to activate target gene expression. We immunostained tissue sections from the cortex of AD patients and asymptomatic age-matched controls to determine localization of pSTAT3_Y705_ within GFAP+ astrocytes. We further labeled for C3, a marker for reactive inflammatory astrocytes, to align with the signature of the iPSC-derived reactive astrocytes after treatment with TNF. We observed pSTAT3_Y705_ signal within the nuclei of GFAP^+^ astrocytes in the AD tissue, while pSTAT3_Y705_ in the asymptomatic tissue was generally situated around the nucleus (Fig. 4D–F and Supplementary Fig 4). Nuclear pSTAT3_Y705_ was also positively correlated with C3 expression (Fig. 4G), highlighting the connections between the in vitro BBB co-culture model and human neuropathology. In addition, mice lacking Stat3 expression in astrocytes (Stat3cKO) exhibited a significant loss in GFAP intensity as well as significantly reduced VCAM-1 expression in brain vasculature relative to wild-type controls (Fig. 5A–C). These outcomes agree with Fig. 1 showing a positive correlation between reactive astrocytes and vascular VCAM-1 expression in human cortical tissue samples.

Based on these data, we revisited the in vitro BBB co-culture model to further clarify direct connections between astrocytic STAT3 activation and BBB dysfunction. Similar levels of astrocytic *STAT3* expression were observed under all culture conditions (Fig. 5D), so we decided to inactivate STAT3 signaling in astrocytes by constitutively overexpressing human Y705 F dominant-negative STAT3 using lentiviral transduction^63^ (Fig. 5E), which significantly reduced VCAM-1 expression and extend TEER longevity in BMEC-like cells after TNF treatment relative to astrocytes transduced with the empty lentiviral backbone (Fig. 5F–H). Since VEGF secretion by astrocytes has been tied to BBB disruption in the inflammatory experimental autoimmune encephalomyelitis (EAE) mouse model^21,64^, we also queried the role of VEGF in the in vitro BBB model. We found that *VEGFA* expression was mostly unchanged by TNF treatment, and knockdown of *VEGFA* in astrocytes in the TNF-treated co-culture system using a CRISPRi strategy (described in more detail below) did not reduce VCAM-1 expression or alter TEER in BMEC-like cells (Supplementary Figs. 5 and 9). Hence, the TNF-STAT3 signaling axis in inflammatory reactive astrocytes contributes to BBB dysfunction, but likely not through VEGF secretion.

### TNF-stimulated reactive astrocytes yield BBB dysfunction through expression of *SERPINA*3

**Fig. 6:**
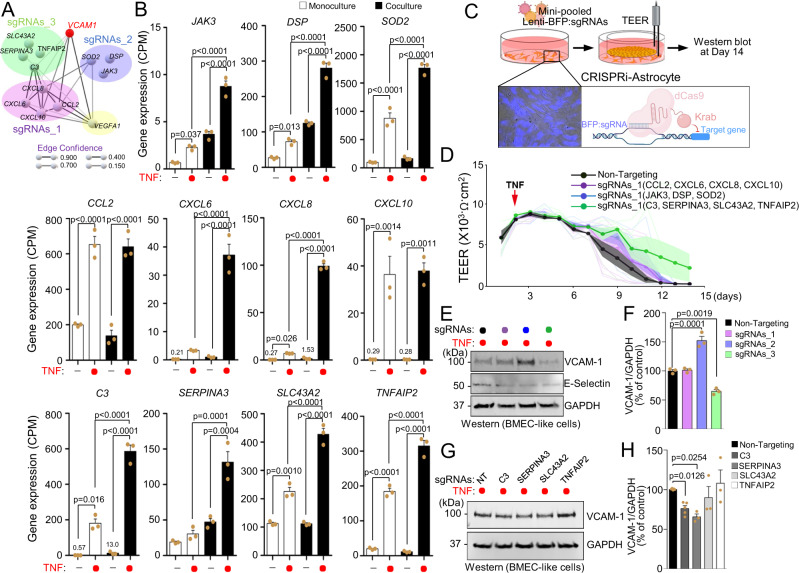
Pooled CRISPRi screen identifies *SERPINA3* expression in astrocytes as a contributor to BBB dysfunction. **A** Network representation connecting *VCAM1* to astrocytic genes predominantly upregulated by TNF in co-culture. Line thickness indicates the edge confidence. The *VCAM1* bait gene is labeled in red. Genes were segregated with colored circles into 4 clusters roughly based on their known functions. **B** Expression levels (CPM) of the selected astrocytic genes of **A**. Data are graphed as mean ± SEM from *n* = 3 biological replicates. Statistical significance was calculated using a one-way ANOVA with Tukey’s post hoc test. **C** Schematic procedure for pooled gene knockdown in CRISPRi-astrocytes and co-culture with BMEC-like cells, followed by TEER measurements and western blot analysis. **D** TEER values in BMEC-like cells in co-culture with astrocytes transduced with pooled sgRNAs. Data are presented as continuous means ± shaded SEMs aggregated from *n* = 3 biological replicates per condition. **E**, **F** Representative western blot and quantification of VCAM-1 expression in BMEC-like cells co-cultured with CRISPRi-astrocytes. CRISPRi-astrocytes were transduced with pooled sgRNAs targeting genes color coded to the clusters in **A**. Data were acquired at day 14 after co-culture. GAPDH was used as a loading control. Data are graphed as mean ± SEM from *n* = 3 biological replicates. Statistical significance was calculated using a one-way ANOVA with Tukey’s post hoc test. **G**, **H** Representative western blot and quantification of VCAM-1 expression in BMEC-like cells co-cultured with CRISPRi-astrocytes. CRISPRi-astrocytes were transduced with a single sgRNA targeting individual genes in the cluster colored in green in **A**. Data were acquired at day 14 after co-culture. GAPDH was used as a loading control. Data are graphed as mean ± SEM from *n* = 3 biological replicates (for non-targeting, *SERPINA3*, *SLC43A2*, and *TNFAIP2*) or *n* = 5 biological replicates (for *C3*). Statistical significance was calculated using a one-way ANOVA with Tukey’s post hoc test.

To further elucidate astrocytic factors that could transduce BMEC inflammation and cause BBB disruption, we used the STRING tool to cluster genes for easier interrogation. The STRING analysis identified 11 genes that were predominantly upregulated in co-cultured astrocytes stimulated with TNF, and these genes were segregated into 3 clusters roughly based on their known functions (Fig. 6A, B). We developed pooled sgRNAs targeting each cluster, which were transduced into CRISPRi astrocytes by lentivirus. In this strategy, iPSCs were modified to express dead Cas9 fused to the KRAB repressor domain and differentiated to astrocytes before receiving the lentivirus. These transduced astrocytes were then co-cultured with BMEC-like cells and treated with TNF, followed by assessments of TEER and VCAM-1 expression (Fig. 6C). Suppression of the chemokine gene cluster (*CXCL10*, *CXCL8*, *CXCL6*, and *CCL2*) did not affect TEER or VCAM-1 expression, which was potentially expected since these genes are mainly involved in recruiting and activating neutrophils^65,66^. In contrast, suppression of the *SOD2*, *DSP*, and *JAK3* cluster increased VCAM-1 expression without affecting TEER. These data are consistent with a previous observation that decreasing SOD2 by TFEB knockdown induced VCAM-1 expression in non-brain endothelial cells^67^. Furthermore, TNF stimulation of STAT3 signaling in astrocytes is typically associated with JAK1 or JAK2 but not JAK3^68,69^, also consistent with our data. In contrast, suppression of *TNFAIP2*, *C3*, *SLC43A2*, and *SERPINA3*, all of which are suggested to be activated by STAT3^58,70–72^, decreased VCAM-1 expression and increased TEER longevity in BMEC-like cells (Fig. 6D–F), which is consistent with the results observed after STAT3 signaling inactivation in astrocytes. Based on these results, we silenced the individual genes from this cluster and re-assessed VCAM-1 expression, which revealed *SERPINA3* as a key effector for mediating BBB damage (Fig. 6G, H). Since *C3* has been previously identified as an astrocyte-derived factor that causes BBB inflammation in mouse models of aging and tauopathy^73^, and we also observed a statistically significant reduction in VCAM-1 expression from *C3* knockdown, we subsequently assessed the effects of simultaneous *SERPINA3* and C3 knockdown. Here, knockdown of both genes yielded a similar VCAM-1 reduction compared to the individual knockdowns and a modest extension of TEER longevity (Supplementary Figs. 6 and 10), suggesting these factors may work independently to cause BBB dysfunction.

### Serpina3n is directly responsible for BBB dysfunction-related molecular changes in cortical explant cultures

**Fig. 7:**
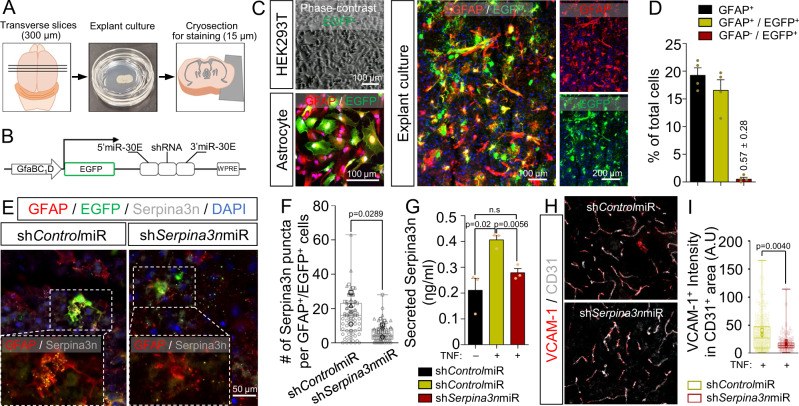
Inhibition of astrocytic Serpina3n expression in cortical explant cultures reduces BBB inflammation associated with TNF treatment. **A** Schematic overview of the cortical explant culture preparation and analyses. **B** Schematic representation of the master AAV vector encoding EGFP and miR−30-shRNA driven by a shortened GFAP promoter. **C** Representative images of EGFP and GFAP expression in HEK293T cells, iPSC-derived astrocytes, and cortical explant cultures after transduction with the AAV vector. **D** Quantification of EGFP and GFAP labeling within the explant cultures. Data are plotted as mean ± SEM for *n* = 4 biological replicates. **E** Representative images of GFAP, EGFP, and Serpina3n expression in cortical explant cultures transduced with the AAV vector encoding either sh*Control*miR or sh*Serpina3n*miR. **F** Quantification of Serpina3n^+^ puncta per GFAP^+^/EGFP^+^ cell from the images in **E**. For each condition, at least 80 EGFP + /GFAP + cells were analyzed. Each quantified cell is represented by a faint data point, and solid datapoints indicate the mean for each biological replicate (*n* = 3). The boxes show the range between the 25th and 75th percentiles, the line within each box indicates the median, and the outer lines extend to 1.5 times the IQR from the box. Statistical analyses were performed with a one-sided *t* test. **G** ELISA quantification of soluble Serpina3n levels in cortical explant cultures after AAV and TNF treatment. Data are graphed as mean ± SEM from *n* = 3 biological replicates. Statistical significance was calculated using a one-way ANOVA with Tukey’s post hoc test. **H** Representative images of CD31 and VCAM−1 expression in cortical explant cultures transduced with the AAV vector encoding either sh*Control*miR or sh*Serpina3n*miR. **I** Quantification of VCAM-1 intensity across total CD31^+^ area (231 vessel segments analyzed in the sh*Control*miR condition and 330 vessel segments analyzed in the sh*Serpina3n*miR condition; *n* = 3 biological replicates per condition). The boxes show the range between the 25th and 75th percentiles, the line within each box indicates the median, and the outer lines extend to 1.5 times the IQR from the box. Faint datapoints indicate individual vessel segments and solid datapoints indicate the mean for each biological replicate. Statistical analyses were performed with a one-sided *t* test.

**Fig. 8:**
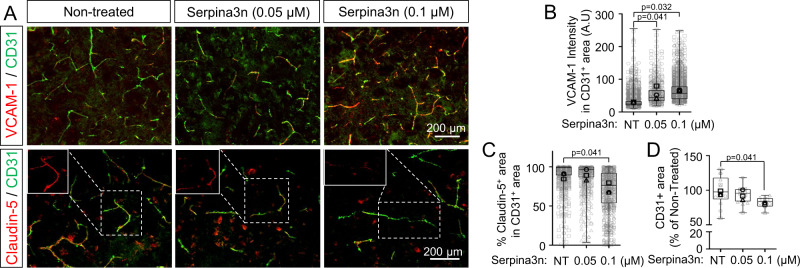
Exogenously administered Serpina3n causes BBB dysfunction-related molecular changes in cortical explant cultures. **A** Representative images of endothelial VCAM-1 expression (upper panel) and claudin-5 expression (lower panel) in cortical explant cultures treated with varying concentrations of recombinant Serpina3n. **B** Quantification of VCAM-1 intensity across total CD31^+^ area. The boxes show the range between the 25th and 75th percentiles, the line within each box indicates the median, and the outer lines extend to 1.5 times the IQR from the box. Faint datapoints indicate individual vessel segments and solid datapoints indicate the mean for each biological replicate (*n* = 3). Statistical significance was calculated using a one-way ANOVA with Tukey’s post hoc test. **C** Quantification of percent claudin-5 coverage within CD31^+^ vessels (0 μM Serpina3n, 1181 vessel segments counted; 0.05 μM Serpina3n, 735 vessel segments counted; 0.1 μM Serpina3n, 673 vessel segments counted; *n* = 3 biological replicates). The boxes show the range between the 25th and 75th percentiles, the line within each box indicates the median, and the outer lines extend to 1.5 times the IQR from the box. Faint datapoints indicate individual vessel segments and solid datapoints indicate the mean for each biological replicate. Statistical significance was calculated using a one-way ANOVA with Tukey’s post hoc test. **D** Quantification of vessel density represented as the area of CD31^+^ vessels against total tissue area. Data are normalized to the non-treated control. The boxes show the range between the 25th and 75th percentiles, the line within each box indicates the median, and the outer lines extend to 1.5 times the IQR from the box. Faint datapoints indicate individual images and solid datapoints indicate the mean for each biological replicate (*n* = 3). Statistical significance was calculated using a one-way ANOVA with Tukey’s post hoc test.

Serpina3n (mouse ortholog of α1ACT) has been previously identified as a reactive astrocyte marker after lipopolysaccharide treatment or middle cerebral artery occlusion^74^, but to our knowledge, no previous studies have linked α1ACT or Serpina3n to BBB dysfunction. To validate the contribution of α1ACT to BBB dysfunction, we transitioned to a mouse cortical explant model (Fig. 7A). First, to permit control over *Serpina3n* expression in astrocytes, we generated an adenovirus-associated virus (AAV)-based system, in which a short GFAP (GfaBC_1_D) promoter drives expression of both EGFP and a miR-30-based short hairpin RNA^75^ (shRNAmiR; Fig. 7B). Astrocyte-specific transgene expression was validated in iPSC-derived astrocytes and mouse cortical explant cultures transduced by the AAV PHP.B capsid^76^ harboring EGFP and shRNAmiR, which demonstrated EGFP expression only to the post-stained GFAP^+^ cells; in contrast, HEK293T cells transduced by the AAV showed no EGFP expression (Fig. 7C, D). Next, we validated the knockdown efficiency in the explant cultures with AAV encoding an shRNA against *Serpina3n* (sh*Serpina3n*miR) or a non-targeting control shRNA (sh*Control*miR). Following TNF treatment, EGFP^+^/GFAP^+^ astrocytes in the sh*Control*miR condition exhibited extensive Serpina3n^+^ puncta, and these puncta were significantly reduced in the sh*Serpina3n*miR condition (Fig. 7E, F). Since Serpina3n is secreted from cells, we additionally performed an ELISA on media collected from each explant culture to validate that silencing *Serpina3n* in astrocytes resulted in depletion of the soluble protein (Fig. 7G). Bolstered by this result, we transduced explant cultures with each AAV and measured VCAM-1 intensity in CD31^+^ vessels, which revealed a significant decrease in VCAM-1 in cultures transduced with sh*Serpina3n*miR relative to sh*Control*miR (Fig. 7H, I). We also treated explant cultures with recombinant mouse Serpina3n, which yielded a dose-dependent increase in VCAM-1 expression within CD31^+^ vasculature, as well as a dose-dependent decrease in claudin-5 tight junction expression within CD31^+^ vasculature and an overall reduction in vascular density (Fig. 8A–D). To connect these results back to STAT3 signaling, we queried Serpina3n expression in the Stat3cKO mouse model. As expected, brain-wide Serpina3n expression was decreased when Stat3 was ablated in astrocytes (Supplementary Fig. 7A, B). Further, *Serpina3n* upregulation in astrocytes due to LPS treatment was significantly attenuated in the Stat3cKO mice (Supplementary Fig. 7C). These results validate outcomes from the in vitro BBB model and provide strong molecular-level evidence that astrocyte-derived Serpina3n may directly cause the neurovascular inflammation and BBB dysfunction related to STAT3 activation.

### Serpina3n causes BBB dysfunction-related molecular changes after direct administration to the brain in vivo

**Fig. 9:**
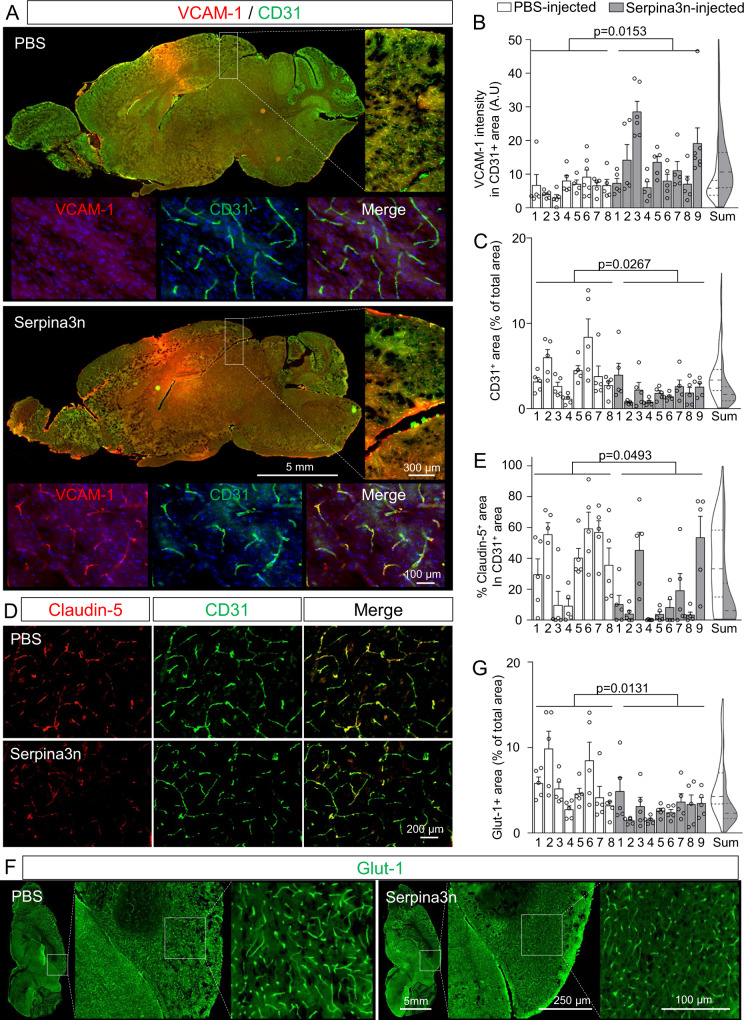
Exogenously administered Serpina3n causes BBB dysfunction-related molecular changes in vivo. Mice received an ICV injection of PBS or Serpina3n, and the entire cohort was euthanized 4 days after injections. For all quantifications, 5–7 regions in each mouse were randomly imaged at a distance of at least 3 mm away from the needle track to minimize contributions from sterile inflammation. **A** Representative sagittal brain section highlighting increased VCAM-1 expression after delivery of Serpina3n. The inset shows that VCAM-1 is localized exclusively to CD31^+^ blood vessels. **B** Quantification of VCAM-1 intensity within total CD31^+^ area in brains of PBS-injected (*n* = 8) versus Serpina3n-injected (*n* = 9) mice. Each data point represents an individual image, and data are bar-graphed as mean ± SEM for each biological replicate. Violin plot with median and 25th–75th percentile represents summarized data. Statistical significance was calculated by a one-sided *t* test. **C** Quantification of vessel coverage by normalizing CD31^+^ area to total tissue area. Each data point represents an individual image, and data are graphed as mean ± SEM for each biological replicate of PBS-injected (*n* = 8) versus Serpina3n-injected (*n* = 9) mouse. Violin plot with median and 25th–75th percentile represents summarized data. Statistical significance was calculated by a one-sided *t* test. **D** Representative images of CD31 and claudin-5 expression in a sagittal brain section. **E** Quantification of claudin-5 coverage within CD31^+^ vessel area. Each data point represents an individual image, and data are graphed as mean ± SEM for each biological replicate of PBS-injected (*n* = 8) versus Serpina3n-injected (*n* = 9) mouse. Violin plot with median and 25th–75th percentile represents summarized data. Statistical significance was calculated by a one-sided *t* test. **F** Representative images of Glut-1 expression in a sagittal brain section. **G** Normalized Glut-1^+^ area. Each data point represents an individual image, and data are graphed as mean ± SEM for each biological replicate of PBS-injected (*n* = 8) versus Serpina3n-injected (*n* = 9) mouse. Violin plot with median and 25th–75th percentile represents summarized data. Statistical significance was calculated by a one-sided *t* test.

Last, we investigated whether Serpina3n could directly induce BBB dysfunction in vivo. To better isolate its effects within the brain, recombinant mouse Serpina3n was administered by intracerebroventricular (ICV) injection. Here, a single dose of Serpina3n was sufficient to induce a significant increase in VCAM-1 expression within CD31^+^ vasculature, relative to PBS vehicle control (Fig. 9A, B). This upregulation in VCAM-1 was widespread, suggesting the effects of Serpina3n are not restricted to a specific brain region, which is consistent with a recent report showing that Serpina3n is ubiquitously induced in reactive astrocytes irrespective of location^77^. In agreement with slice culture experiments, the expression of claudin-5 was also decreased within CD31^+^ vasculature due to Serpina3n injection (Fig. 9D, E). We also noted a reduction of vasculature density due to Serpina3n treatment, as measured by total area of CD31^+^ and Glut-1^+^ blood vessels (Fig. 9C, F, G). Last, we performed a tracer extravasation assay where, after ICV injection of PBS or Serpina3n, a 3 kDa fluorescent dextran was injected intraperitoneally and its levels in brain were quantified relative to serum. Here, we did not observe a significant increase in dextran in the brain as a function of exogenously administered Serpina3n (Supplementary Fig. 8). This may reflect size-selective BBB disruption that was not captured by the dextran or the previously observed reduction in vascular density, which could have decreased the capillary surface area available for dextran transfer and thereby influenced the permeability measurement in unforeseen ways. These issues will require further experimentation to clarify. From our collective data, we conclude that the effects of Serpina3n observed in vivo are consistent with the outcomes in slice cultures, further highlighting a role for Serpina3n in BBB dysfunction-related molecular changes.

## Discussion

Recently, it has been proposed that proinflammatory conditions generate subsets of inflammatory reactive astrocytes that may be active participants in neurodegenerative disease progression. Inflammation in brain endothelial cells is also a common feature of age-related neurodegenerative diseases. However, a detailed understanding of the molecular basis and interactions between subtypes of reactive astrocytes and brain endothelial cells has remained largely unknown. Progress in this area has been limited by the heterogeneity of reactive astrocytes in vivo, as well as the absence of effective in vitro systems that permit long-term quiescent co-cultures of astrocytes and brain endothelial cells that can be perturbed in a controlled manner. Indeed, in vitro cultures of astrocytes and brain endothelial cells have historically relied on serum, an inducer of astrocyte reactivity^74,78^, which prevented defined cause-and-effect studies of astrocyte/endothelial crosstalk. Herein, we focused on a simplified human BBB model generated from iPSCs, which offers a scalable system that also permits tight control over experimental conditions. The differentiation of iPSCs to BMEC-like cells and astrocytes has been iterated over the past decade, and a recent step forward that enabled this current study was our prior establishment of a serum-free protocol yielding BMEC-like cells with representative BBB characteristics. This allowed us to use a variety molecular tools and analyses to understand BBB alterations to astrocyte reactivity.

Previous studies have shown that iPSC-derived BMEC-like cells do not express VCAM-1 on the cell surface after TNF stimulation^40^, in contrast to primary BMECs that readily upregulate VCAM-1^79^. After validating that we could convert iPSC-derived astrocytes to a GBP2^+^ inflammatory reactive state, we studied whether astrocytes could influence BMEC responses to TNF. We ultimately determined that active astrocyte/endothelial crosstalk was necessary for TNF to disrupt passive BBB function and yield upregulation of VCAM-1 on the BMEC-like cells. These findings suggested BBB inflammation is not fully cell autonomous, a notion supported by other studies^80,81^.

Activation of STAT3 signaling has been suggested as a central axis for a subpopulation of reactive astrocytes in CNS disorders^60,61,82,83^. For example, in mouse models, STAT3 signaling in reactive astrocytes has been tied motor outcomes in stroke^84^, and plaque burden and memory deficiencies in AD^60^. In human patients, STAT3 activation in astrocytes regulates cancer metastasis to brain^85^. The severity and progression of many brain diseases are also linked human neurovascular dysfunction^86,87^, which suggested links between STAT3 activation in astrocytes and BBB dysfunction. Our in vitro data, showing rescue of BBB properties through inhibition of STAT3 and its downstream targets in astrocytes, as well as our human tissue assessments, support this link. Our merged investigations into STAT3-dependent astrocyte reactivity and BBB dysfunction further allowed us to identify α1ACT/Serpina3n as a BBB-damaging factor. Historically, Serpina3n and α1ACT have been associated with astrocytes in mouse models of AD^88^ and postmortem human tissue^89^, respectively, and they are believed to play a role in amyloid beta aggregation^90^. More recent studies have linked Serpina3n to factors associated with BBB dysfunction, without providing direct links between Serpina3n and BBB dysfunction. For example, the APOE4 genotype yields increased expression of Serpina3n in humanized APOE mice relative to APOE3 and APOE2^91^, and in separate work, APOE4 humanized mice exhibited increased BBB disruption relative to APOE3 and APOE2 mice through a cyclophilin A-dependent inflammatory cascade^92^. APOE4 has also recently been identified as a driver of BBB dysfunction and cognitive impairment in human subjects independent of amyloid and tau pathology^93^, which could potentially be linked to α1ACT. Aging and intraperitoneal injection of lipopolysaccharide, which yield loss of BBB function, also produce an increase in Serpina3n expression in astrocytes across many different brain regions^18,77,94^. Hence, we speculate that astrocyte-derived Serpina3n/α1ACT could be an unrecognized driver of BBB dysfunction in many diseases. Indeed, α1ACT was recently identified as a potential biomarker for progressive for multiple sclerosis due to its elevated levels in cerebrospinal fluid^95^, which could be connected to increased VCAM-1 expression at the BBB to license immune cell entry into the brain.

One limitation of our study is that we did not examine the role of specific subtypes of reactive astrocytes on BBB inflammation and dysfunction. Recent work suggests this will be a critical consideration moving forward. For example, reactive astrocytes were recently shown to support vascular repair and remodeling after stroke, with worsened outcomes if reactive astrocytes were ablated^96^. This animal model of stroke utilized photoablation rather than artery occlusion, which is the stroke model in which Serpina3n was identified as a reactive astrocyte marker^74^, and *Serpina3n* was not highlighted in the photoablation datasets. However, a recent single-cell transcriptomic analysis of AD and non-AD aged individuals identified SERPINA3/α1ACT in one out of six astrocytic clusters^97^. Hence, the specificity of the injury, its anatomical location, and the type of astrocyte it activates may be key for induction of the STAT3/Serpina3n damage pathway that we have identified. We note that the reactive astrocyte state characterized in our study aligns with the “STAT3-dependent reactivity” state identified by Kun et al, which is defined by an acute phase inflammatory response to IL-1α, TNFα, and C1q leading to upregulation of similar genes^72^; however, this state also likely represents a spectrum of reactive astrocyte subtypes, particularly when considering that our primary metric for assessing reactive astrocyte identity was expression of the pan-reactivity marker GBP2. Another recent study also showed that differential reactive astrocyte expression of *Serpina3n* occurs in some but not all animal disease models^98^, and it remains to be seen how this outcome could be related to BBB dysfunction in different diseases. We suggest that future work will need to assess connections between reactive astrocyte subsets and BBB dysfunction in more nuanced in vitro models, animal models, and human tissue cohorts. Such efforts will be necessary to build on our findings and continuously shape an understanding of how reactive astrocyte-BBB crosstalk contributes to human neurodegeneration.

## Methods

### Compliance statement

Human brain tissue collection procedures were approved by the Institutional Review Board at Vanderbilt University Medical Center, and written consent for brain donation was obtained from patients or their surrogate decision-makers. At Vanderbilt University, all animal procedures were performed in accordance with a protocol approved by the Institutional Animal Care and Use Committee. At the University of California Los Angeles, all animal procedures were conducted according to protocols approved by the Chancellor’s Animal Research Committee of the Office for Protection of Research Subjects. Mice were housed in a 12-hour light/dark cycle and allowed ad libitum access to food and water.

### iPSC maintenance

The CC3 iPSC line was used for all experiments unless otherwise stated^99^. Undifferentiated iPSCs were maintained in E8 medium on 6-well plates coated with growth factor reduced Matrigel (Corning). iPSCs were passaged with Versene (Thermo Fisher Scientific) upon reaching 60–80% confluency.

### iPSC differentiation to astrocytes

To reduce variability in astrocyte generation, manually isolated neural progenitors were used as the starting population. As shown in Fig. 2A, iPSCs were dissociated with Versene and seeded into 6-well low-attachment plates to form embryoid bodies (EBs) at a density of 4 × 10^5^ cells/well in E6 medium supplemented with 100x N2 (Invitrogen; E6N2) and 10 μM Y27632 (Tocris). Neural differentiation in the EBs was achieved by dual inhibition of SMAD signaling for 7 days^100^. EBs were then seeded onto growth factor reduced Matrigel-coated plates in E6N2 containing 1 μg/ml laminin (Sigma Aldrich). After another 7 days (Day 0), neural progenitor cells were manually isolated from rosettes and expanded as neurospheres in a suspension culture for 7 days in E6N2 supplemented with 50× B27 lacking retinoic acid (Invitrogen) and 20 ng/ml FGF2 (Peprotech). Then, the neurospheres were dissociated into single cells with TrypLE^TM^ (Thermo Fisher Scientific) and seeded onto growth factor reduced Matrigel-coated 6-well plates in E6N2 supplemented with 50x B27 lacking retinoic acid, 10 ng/ml BMP4 (Peprotech) and 20 ng/ml FGF2 for directed astroglial differentiation^101^. Medium changes were performed every 48 h, and cells were passaged upon reaching approximately 80% confluency. Astrocytes were seeded for co-culture at a ratio of 1 well of a 6-well plate to 6 wells of a 12-well plate and maintained in E6 medium containing 10 ng/ml CNTF (Peprotech) and 10 ng/ml EGF (Peprotech) until initiation of co-culture.

### iPSC differentiation to BMEC-like cells

Differentiations were carried out as previously described^35^. iPSCs were dissociated with Accutase and seeded onto growth factor reduced Matrigel-coated plates in E8 medium containing 10 μM Y27632 at a density of 15,000 cells/cm^2^. Differentiation was initiated 24 h after seeding by changing to E6 medium, with daily medium changes for 4 days. Next, cells were expanded with serum-free basal endothelial cell medium (EC medium) supplemented with 50x B27 (Thermo Fisher Scientific), GlutaMAX^TM^ (Thermo Fisher Scientific), 10 μM retinoic acid (Sigma Aldrich), and 20 μg/ml FGF2 for 2 days without media change. Following this treatment, cells were collected by a 20 min incubation in Accutase and seeded onto Transwell filters (1.1 cm^2^ polyethylene terephthalate membranes with 0.4 μm pores; Fisher Scientific) coated with a mixture of 400 μg/ml collagen IV (Sigma Aldrich) and 100 μg/ml fibronectin (Sigma Aldrich). The following day, cells were switched to EC medium lacking FGF2 and RA. If co-culture was being initiated, filters were transferred to 12-well plates containing astrocytes and the same medium was utilized. Starting at this time, transendothelial electrical resistance (TEER) was measured using STX2 chopstick electrodes and an EVOM2 voltameter (World Precision Instruments) and approximately every 24 h thereafter.

### Cytokine treatments on astrocytes and BMEC-like cells

To prepare conditioned medium, astrocyte cultures at 80% confluence in 6-well plates (in E6 medium containing BMP4 and FGF2) were treated with vehicle control or IL-1a (5 ng/ml; Peprotech), TNF (10 ng/ml; Peprotech), and C1a (500 ng/ml; MyBioSource) for 72 h. After three washes with DPBS, cells were placed in minimal conditioning medium containing phenol-red-free DMEM/F12 (Thermo Fisher Scientific) and Glutamax (Gibco). Spent media were collected after 3 days, with cell debris removed by centrifugation at 1800 × *g*, 4 °C for 5 min. The supernatants were concentrated in centrifugal concentrators (Millipore Sigma) with a size cutoff filter of 3 kDa. Protein concentration was determined by a BCA assay (Thermo Fisher Scientific), and the solutions were added to BMEC-like cells at 100 μg/ml. For direct treatment of cytokines on either BMECs in monoculture or co-culture with astrocytes, a full media change with vehicle control or cytokine (5 ng/ml IL-1a, 10 ng/ml TNF, 500 ng/ml of C1q, or 10 ng/ml of IFNγ) was performed a day after seeding of BMEC-like cells onto Transwell filters as described above.

### Generation of adeno-associated virus (AAV)

Plasmid pAdDeltaF6 was a gift from James Wilson (Addgene plasmid #112867). pUCmini-iCAP-PHP.B was a gift from Viviana Gradinaru (Addgene plasmid #103002). Plasmid GFAP(short)-shRNAmiR was constructed by VectorBuilder Inc. The shRNAmiR sequences in transfer vectors were incorporated into AAV particles using pUCmini-iCAP-PHP.B and pAdDeltaF6. Virus was produced by transfecting all plasmids at a 1:1:1 molar ratio (30 μg total DNA) into HEK293T cells with Lipofectamine3000 (Invitrogen). Viral particles were collected at 72 h by filtering spent medium through a 0.45 μm cellulose acetate filter (Millipore Sigma), then concentrating the supernatant with AAVpro® concentrator (TaKaRa).

### Generation of lentivirus

Plasmid EF.STAT3DN.UBC.GFP, which contains the cDNA encoding for the dominant-negative form of human STAT3 (STAT3DN), was a gift from Linzhao Cheng (Addgene plasmid #24984). Plasmid pMK1334 for lentiviral delivery of sgRNA was a gift from Martin Kampmann (Addgene plasmid #127965). Sequences in transfer vectors were incorporated into lentivirus using the lentiviral packaging system (pMD2.G and psPAX2), which was a gift from Didier Trono (Addgene plasmid #12259 and #12260) in HEK293T cells using Lipofectamine 3000 (Invitrogen). After 48 and 72 h, spent medium was collected and filtered through a 0.45-μm cellulose acetate filter before concentrating the supernatant using Lenti-X^TM^ concentrator (TaKaRa).

### RNA sequencing and data analysis

All samples were collected at the day 5 time point indicated in Fig. 2J, regardless of monoculture or co-culture. Total RNA was isolated using TRIzol (Sigma Aldrich). RNA was further purified with a Qiagen RNeasy Plus mini kit (Qiagen) and submitted to the Vanderbilt Technologies for Advanced Genomics (VANTAGE) core for sequencing using an Illumina NovaSeq6000. Samples were prepared for sequencing using the TruSeq RNA sample prep kit (Illumina) to prepare cDNA libraries after ribosome depletion. Raw sequencing reads were obtained for the paired-end samples. FASTQ reads were mapped to the human genome (hg19, Feb. 2009 GRCh37) by HISAT2 (2.2.0). EdgeR (3.30.3) and limma^102^ software packages were used to measure differential gene expression with genes that achieved a count per million mapped reads (CPM). Any genes not considered to be detected (CPM <4) were removed. Adjusted *p* values (<0.05) were utilized for functional enrichment analysis with the WEB-based Gene SeT AnaLysis Toolkit (WebGestalt)^103^. For interactome prediction between astrocytic secretome and endothelial receptome, the STRING database^104^ was used with combined edge confidence scores of >0.7 of the human interactome version 11.0b. Network figures were created using Origene2021 where nodes refer to genes through identified protein-protein interaction networks. For an unbiased analysis of potential differentially regulated pathways in astrocytes, differentially expressed genes (FDR < 0.1) and corresponding expression values were uploaded into the Ingenuity Pathway Analysis system (http://www.ingenuity.com).

### qPCR analysis

Total RNA was extracted with TRIzol and converted to cDNA using a Superscript III First-Strand kit (Invitrogen). qPCR was performed on a BioRad CFX96 Thermocycler with TaqMan primers against *VEGFA*, *VCAM1*, and *GAPDH* (Thermo Fisher Scientific). Samples were analyzed by normalizing expression levels to *GAPDH* and relative quantification was performed using the standard 2-ΔΔC_t_ method.

### STAT3 signaling inhibition in iPSC-derived astrocytes

For experiments involving overexpression of dominant-negative STAT3 _Y705F_, freshly plated iPSC-derived astrocytes (10^6^ cells per 6-well plate) were incubated for three days with lentiviral particles expressing dominant-negative STAT3_Y705F_ in E6 medium containing 10 ng/ml BMP4 and 10 ng/ml EGF. Astrocytes were expanded and maintained by changing medium every 48 h until initiation of co-culture with BMEC-like cells. Empty viral particles were used as a control. Expression of EGFP in astrocytes was confirmed on an epifluorescent microscope before proceeding with experiments.

### CRISPR interference (CRISPRi) experiments

WTC11 iPSCs with stably integrated CRISPRi machinery and dox-inducible NFIA-SOX9^72^ were dissociated to a single-cell suspension with Accutase and then replated at 7500 cells/cm^2^ in Matrigel-coated 10-cm or 15-cm dishes with DMEM/F12 basal medium supplemented with 50× B27, 100× N2, and 20 μg/ml FGF2. The next day, media was changed to ScienCell Astrocyte Media (ScienCell Research Laboratories) containing 2 μg/ml doxycycline (Millipore Sigma) to initiate astrocyte differentiation. A full media exchange was conducted every other day, with doxycycline maintained at 2 μg/ml throughout the differentiation process. In 5–6 days, after confluency was reached, the cultures were dissociated and split 1:8. Expansion of the cultures with the same split ratio was continued until day 20 of differentiation. The final CRISPRi-astrocytes were then characterized as described previously^72^. Prior to co-culture with BMEC-like cells, CRISPRi-astrocytes were incubated with lentiviral particles for 3 days and then passaged once with media exchanges every other day. The following sgRNAs were cloned into the pMK1334 vector before lentiviral production:

*JAK3* (GACCTAGCGGGCAGGGACCC)

*DSP* (GAACTCGGACGGCTACTGGT)

*SOD2* (GGGCGCAGGAGCGGCACTCG)

*CCL2* (GAGCAGCCAGAGGAACCGAG)

*CXCL6* (GGTGGAGGCGCGGAGACTGG)

*CXCL8* (GAGAGCCAGGAAGAAACCAC)

*CXCL10* (GCTGGAGGTTCCTCTGCTGT)

*C3* (GTGCAGGGTCAGAGGGACAG)

*SERPINA3* (GAAAAGGCACAGGGAATCAG)

*SLC43A2* (GAGATCAGCGCCCAGACCTA)

*TNFAIP2* (GAATGGACGTGTGGAAGCGG)

*VEGFA* (GGGGCAGCCGGGTAGCTCGG).

### Human postmortem brain tissue

A diagnostic postmortem evaluation was performed to confirm the presence of Alzheimer’s disease or cerebral amyloid angiopathy following the National Alzheimer’s Coordinating Center (NACC) Neuropathology Data Form. The tissue was flash-frozen in liquid nitrogen at the time of donation. De-identified brain tissue from a patient with unspecified non-surgical convulsive seizures was obtained via Vanderbilt University Medical Center Cooperative Human Tissue Network.

### Animal experiments

#### Slice culture preparation and use

P6 C57BL/6J mice (both male and female pups) were sacrificed by cervical dislocation. Brains were removed, mounted, and cut into 300 μm coronal slices. Transverse slices of 300 μm were made on a vibrating blade microtome (Leica) in cold Hank’s Balanced Salt Solution supplemented with 100 U/ml penicillin/streptomycin, 10 mM HEPES, and 4.5 mM sodium bicarbonate. Each slice was transferred to a membrane insert (PICMORG50, Millipore Sigma) in a 35 mm culture dish with 1.2 ml of DMEM supplemented with 3.5 g/l D-glucose, 0.2 mM Glutamax, 10 mM HEPES, 25 U/ml penicillin/streptomycin, 50x B27 Plus, and 0.1 mM D-Luciferin sodium salt (Tocris). For knockdown of Serpina3n, slice cultures were transduced with AAV particles at the onset of culture. AAV particles harbored an shRNA sequence targeting *Serpina3n* (AACGGGTAGTGCCCTGTTTATT) or the original shRNA sequence targeting LacZ as a negative control (TGTCGGCTTACGGCGGTGATTT). Three days later, the slice cultures were stimulated for another three days with 10 ng/ml TNF prior to analysis. For Serpina3n dose-response assays, recombinant mouse Serpina3n (R&D Systems), at a concentration of 50 or 100 ng/ml, was added at the onset of culture and incubated for four days. For all experiments, slice chambers were sealed with a coverslip and maintained in a humidified incubator.

#### Evaluation of BBB integrity via immunostaining after intracerebroventricular injection of Serpina3n in wild-type mice

Sixteen-week-old wild-type C57BL/6J male mice (Jackson Laboratories) were randomly assigned to the control (PBS) and Serpina3n-injected groups. Under isoflurane anesthesia, mice received a stereotactic injection directly into the ventricle at coordinates of AP = −0.3 mm, ML = −1 mm, and DV = −3 mm. After injections, mice were immediately returned to prewarmed cages. The first group received an injection of 5 μl of sterile saline solution and the second group received an injection of 10 ng/μl Serpina3n solution (50 ng in 5 total μl). Four days after surgery, mice were transcardially perfused with PBS and sacrificed for brain extraction and histology.

#### Evaluation of BBB integrity via dextran extravasation after intracerebroventricular injection of Serpina3n in wild-type mice

Eighteen-week-old wild-type C57BL/6J male mice were randomly assigned to the control (PBS) and Serpina3n-injected groups. Under isoflurane anesthesia, mice received a stereotactic injection directly into the ventricle at coordinates of AP = −0.3 mm, ML = −1 mm, and DV = −3 mm. After injections, mice were immediately returned to prewarmed cages. The first group received an injection of 5 μl of sterile saline solution and the second group received an injection of 10 ng/μl Serpina3n solution (50 ng in 5 total μl). Four days after surgery, 100 µl of 2 mM tetramethyl rhodamine (TMR) dextran (3 kDa; Thermo Fisher Scientific: D3308) was administered intraperitoneally (IP). Two additional age matched mice were used as shams and were not given dextran IP. After 2 h, mice were anesthetized and perfused transcardially with 1× PBS. During surgery, 200–300 µl of blood was collected from the animal by puncturing the right atrium before PBS perfusion. Post perfusion, brain and kidneys were immediately extracted, visually checked for perfusion quality, and frozen on dry ice. For homogenization, tissue was thawed on ice and homogenized in 1× PBS using a Qiagen TissueLyser LT (50 oscillations/s for 10 min). Once tissue was homogenized, samples were centrifuged for 20 min at 15,000 × *g* and 4 °C, and supernatant was collected for florescence measurements. Post collection, blood samples were centrifuged for 15 min at 1300 × *g* and 4 °C, and supernatant was collected as serum for fluorescence measurements. Serum was diluted in 1× PBS (30 µl PBS:20 µl serum), and samples were loaded in duplicate into a black, flat bottom, 384-well microplate. Florescence was acquired using the BioTek Cytation 3 imaging reader, where excitation/emission was set to 550/580 nm, and gain was optimized to 85. Duplicate wells were averaged, and mean sham tissue and serum relative fluorescence units (RFU) were subtracted from each respective tissue type. Permeability index was automatically calculated by Python codes as: Permeability Index (ml/g) = (Tissue RFUs/g tissue weight)/(Serum RFUs/ml serum)^105^.

#### Evaluation of astrocyte reactivity and BBB integrity by immunostaining in Stat3-cKO mice

The *Stat3* gene was conditionally deleted from astrocytes in the well-characterized 73.12 *mGfap*-Cre-*Stat3*-LoxP mouse strain on a C57BL/6 background. For comparisons to mice with Stat3 expression intact, 73.12 GFAP-Cre and Aldh1L1-CreERT mice on a C57BL/6 background were used as negative controls. Male and female mice were used from 2 to 4 months of age. After terminal anesthesia by barbiturate overdose, mice were transcardially perfused with 4% paraformaldehyde (Electron Microscopy Sciences), and brains were removed for downstream processing.

### Immunostaining

Cryosectioned tissues, slice cultures, and cultured cells were fixed with 4% paraformaldehyde and processed for immunofluorescence staining. Samples were permeabilized with 0.3% Triton X-100 in PBS, blocked with 10% goat serum in PBS, and incubated at 4°C overnight with the following primary antibodies: mouse anti-C3 (1:200 dilution, 846302, Biolegend), rabbit anti-CD31 (1:200 dilution, RB-10333, Thermo), mouse anti-CD44 (1:1000 dilution, AB6124, Abcam), anti-Claudin-5-Alexa Fluor 488 (1:1000 dilution, 352488, Invitrogen), mouse anti-GBP2 (1:100 dilution, LS-B12172-50, LSBio), rabbit anti-GFAP (1:300 dilution, Z0334, Dako), mouse anti-GFAP (1:300 dilution, MAB360, Millipore), chicken anti-GFAP (1:300 dilution, SKU: GFAP, Aves), goat anti-GFP (1:1000 dilution, 600-141-215, Rockland), mouse anti-Glut1 (1:1000 dilution, FAB1418G, R&D systems), mouse anti-occludin (1:500 dilution, 33-1500, Thermo Fisher Scientific), rabbit anti-pSTAT3(y705) (1:300 dilution, 9145S, Cell Signaling), goat anti-Serpina3n (1:200 dilution, AF4709-SP, Abcam), rabbit anti-VCAM-1 (1:200 dilution, ab134047, Abcam), mouse anti-VCAM-1 (1:200 dilution, sc-13160, Santa Cruz), goat anti-VE-cadherin (1:300 dilution, AF938, R&D Systems). The following day, after washing with PBS, for unconjugated antibodies, immunostaining was completed by a 1-hour room temperature incubation with secondary antibody (donkey anti-rabbit, anti-mouse, anti-chicken, or anti-goat Alexa Fluor 488, Alexa Fluor 546, or Alexa Fluor 647; 1:1000 dilution; Thermo Fisher Scientific). Tissue sections were mounted with the anti-fade Fluoromount-G medium containing 4’,6-diamidino-2-phenylindole dihydrochloride (DAPI; Southern Biotechnology), while cultured cells were labeled with DAPI (Thermo Fisher Scientific) diluted in PBS for 10 min. Images were acquired with a Zeiss LSM700 T-PMT confocal microscope or a Leica DMi8 epifluorescence microscope. In select cases, images were analyzed using customized scripts for the macro function of ImageJ software (1.53c).

### Western blot analysis

BMEC-like cells were lysed with RIPA buffer (Thermo Fisher Scientific) supplemented with protease inhibitor (Sigma Aldrich). Lysed protein was separated on 12% SDS-PAGE gels and transferred onto nitrocellulose membranes. Blots processed with blocking solution were incubated with antibodies against VCAM-1 (ab134047, Abcam, 1:1000 dilution), E-Selectin (AF724, R&D systems, 1:1000 dilution), and GAPDH (D16H11, Cell Signaling, 1:5000 dilution). The membranes were then washed and incubated with LI-COR fluorophore-conjugated second antibodies (926-68024, 926-32214, 926-68022, 926-32210, and 926-32211). Afterward, western blots were visualized using an Odyssey® Fc (LI-COR).

### Enzyme-linked immunosorbent assay detection of Serpina3n

Slice cultures were treated with AAV particles for 3 days, followed by TNF or vehicle control for 3 days, then spent medium was collected. A Serpina3n-specific sandwich ELISA kit (Aviva Biosciences) was used according to the manufacturer’s recommendation. Briefly, spent media were allowed to react with a capture anti-Serpina3n antibody on the surface of 96-well ELISA plates for 2 h at room temperature. A second anti-Serpina3n antibody was used to detect immobilized Serpina3n, followed by incubation with an HRP-conjugated antibody and detection at 450 nm. Quantification was achieved using a standard curve.

### Reporting summary

Further information on research design is available in the Nature Research Reporting Summary linked to this article.

## Supplementary information

Supplementary Information

Reporting Summary

## Data Availability

RNA sequencing data have been uploaded to ArrayExpress under the accession number E-MTAB-11468. The human genome (hg19, Feb. 2009 GRCh37) is available from the NCBI Dataset Genome page. Source data are provided with this paper.

## Source data

Source Data
