# Supplementary material for: Reactive astrocytes transduce inflammation in a blood-brain barrier model through a TNF-STAT3 signaling axis and secretion of alpha 1-antichymotrypsin
Source: Nat Commun. 2022 Nov 2;13:6581. doi: 10.1038/s41467-022-34412-4 (PMC9630454; doi:10.1038/s41467-022-34412-4)
Supplement: Supplementary file 1 — Supplementary Information [file 41467_2022_34412_MOESM1_ESM.pdf]

Supplementary Information

Reactive astrocytes transduce inflammation in a blood-brain barrier model through a TNF-STAT3 signaling axis and secretion of alpha 1-antichymotrypsin

Hyosung Kim, Kun Leng, Jinhee Park, Alexander G. Sorets, Suil Kim, Alena Shostak, Rebecca J. Embalabala, Kate Mlouk, Ketaki A. Katdare, Indigo V. L. Rose, Sarah M. Sturgeon, Emma H. Neal, Yan Ao, Shinong Wang, Michael V. Sofroniew, Jonathan M. Brunger, Douglas G. McMahon, Matthew S. Schrag, Martin Kampmann, and Ethan S. Lippmann

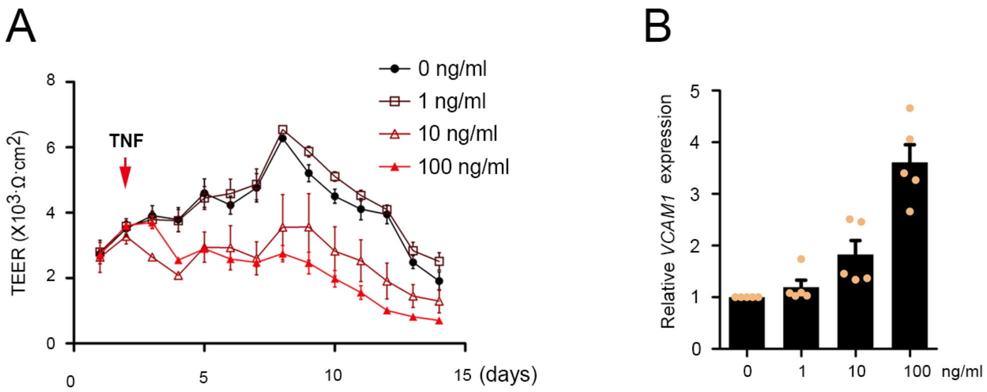

Supplementary Figure 1. Dose-dependent effects of TNF on cocultures of iPSC-derived BMEC-like cells and astrocytes.

(A) Representative TEER values in BMEC-like cells as a function of TNF dose. Data points represent mean ± SEM from duplicate Transwell filters per condition. Trends were confirmed across biological n=3.

(B) *VCAM1* expression in BMEC-like cells at day 14 as a function of TNF dose. Data are normalized to the untreated control and graphed as mean ± SEM from n=3 biological replicates.

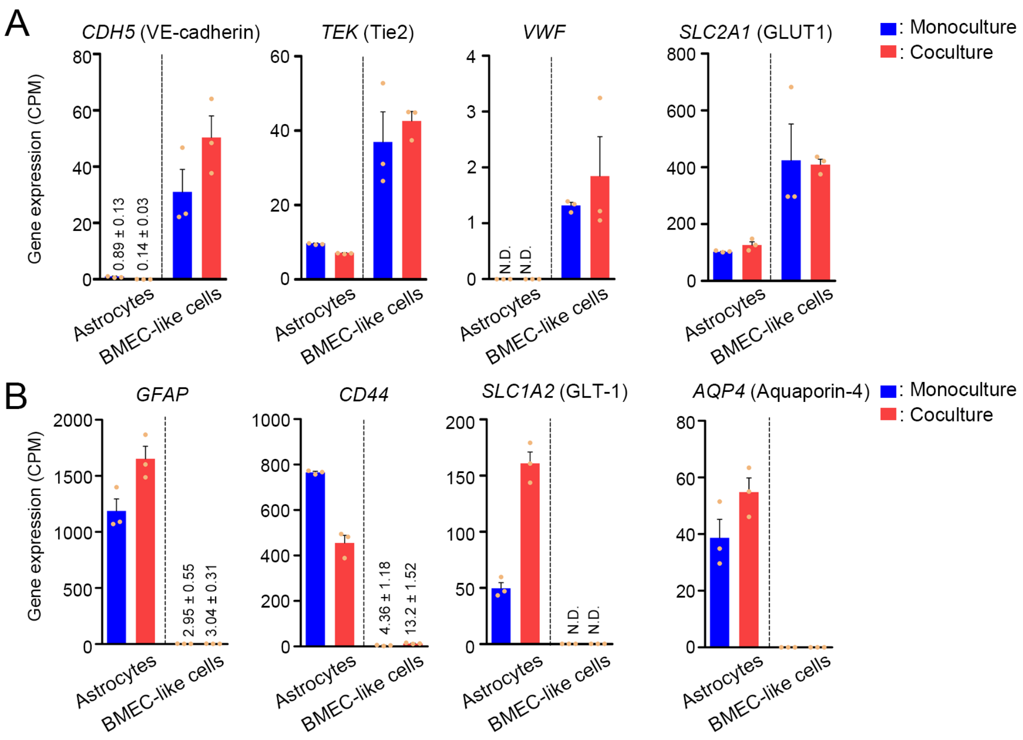

Supplementary Figure 2. Validation of astrocytic and endothelial cell identity by cell-type-specific gene expression.

Contrasted expression of endothelial-enriched (panel A) and astrocyte-enriched (panel B) genes in astrocyte and BMEC-like cells in monoculture or coculture. Data are graphed as mean ± SEM from n=3 biological replicates. N.D., not detected.

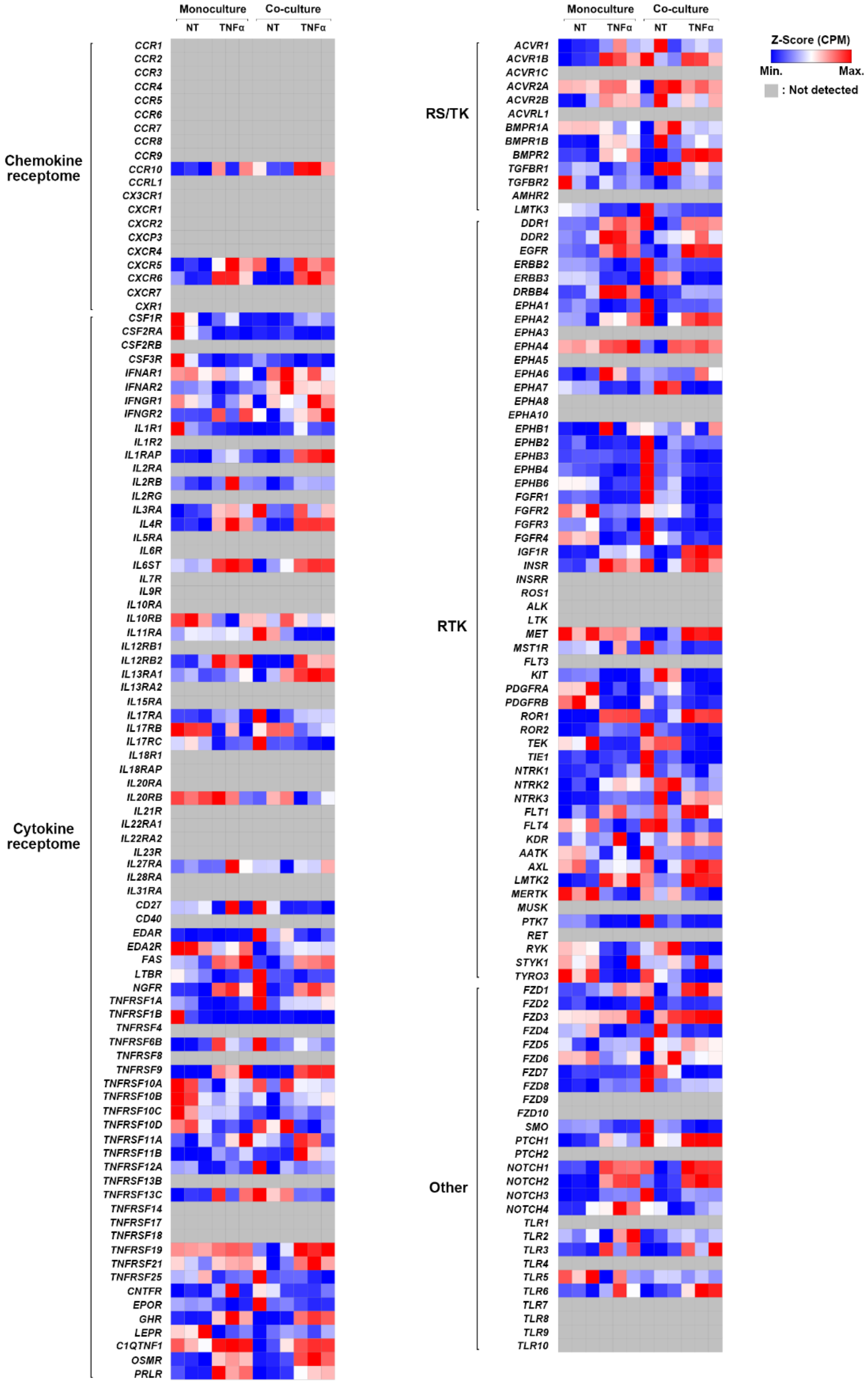

Supplementary Figure 3. Receptome gene classifications in BMEC-like cells.

Heat map showing gene expression of 194 human transmembrane receptors across all BMEC-like cell conditions. The gene sets are based on Kang et al., 2013[1]. The color of the heat map represents the CPM value for each gene.

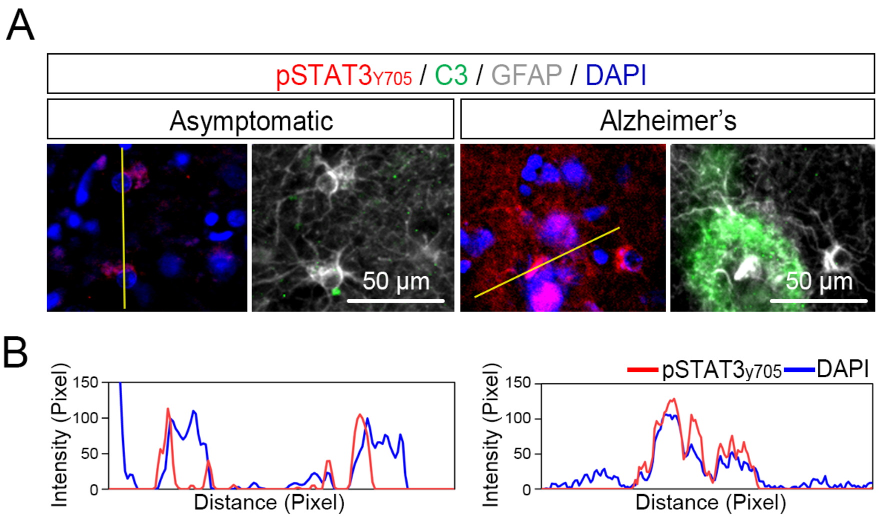

Supplementary Figure 4. Intensity profile analysis for localization of pSTAT3$_{Y705}$ in human brain tissue.

(A) Representative images of pSTAT3$_{y705}$, C3, and GFAP expression in an asymptomatic patient (left) versus a patient diagnosed with Alzheimer's disease (right). Trends were confirmed across n=3 biological replicates per condition.

(B) Graphs showing one-dimensional intensity profile corresponding to each yellow line in panel A. Images were analyzed using ImageJ software.

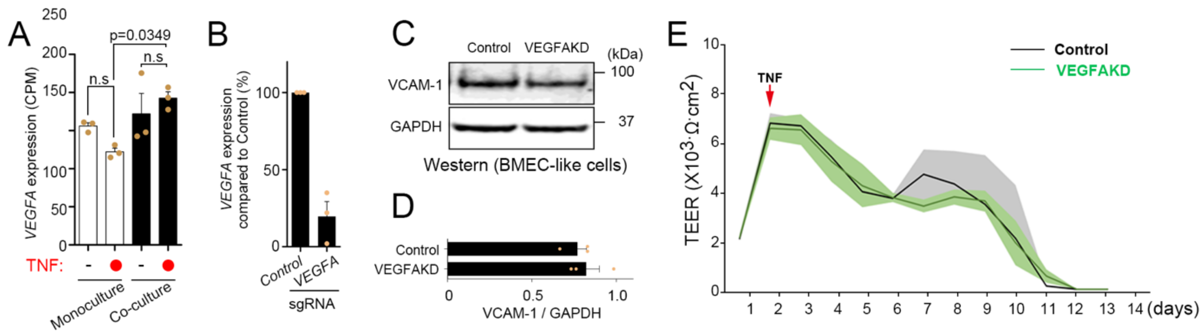

Supplementary Figure 5. Effect of astrocytic *VEGFA* knockdown on BBB properties.

(A) Expression levels (CPM) of *VEGFA* in across all astrocyte conditions. Data are graphed as mean ± SEM from n=3 biological replicates (one-way ANOVA with Tukey's post hoc test).

(B) Relative *VEGFA* expression in CRISPRi-astrocytes transduced with sgRNA targeting *VEGFA*. Data are presented as mean ± SEM from n=3 biological replicates.

(C-D) Representative western blot (panel C) and quantification (panel D) of VCAM-1 expression in BMEC-like cells cocultured with CRISPRi-astrocytes. Samples were isolated on day 14 of coculture. Data are presented as mean ± SEM from n=3 biological replicates.

(E) TEER values in BMEC-like cells in coculture with CRISPRi-astrocytes transduced with sgRNAs. Data are presented as continuous means ± shaded SEMs aggregated from n=3 biological replicates per condition.

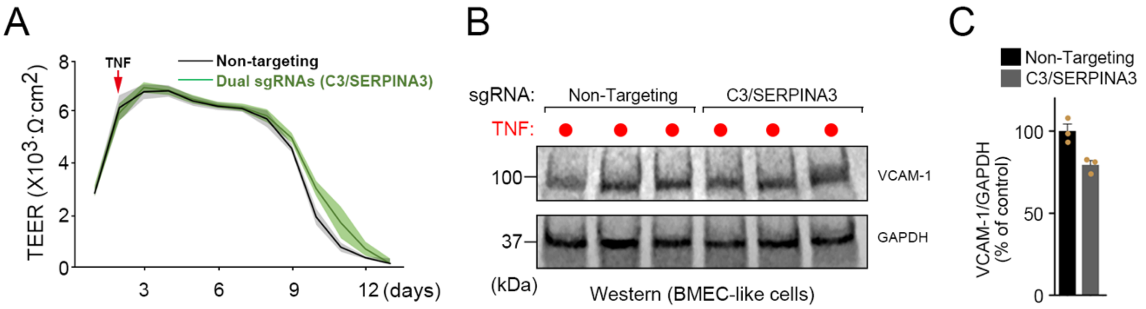

Supplementary Figure 6. Effect of simultaneous *SERPINA3* and *C3* knockdown on BBB properties.

(A) TEER values in BMEC-like cells in coculture with CRISPRi-astrocytes transduced with dual sgRNAs targeting *C3* and *SERPINA3*. Data are presented as continuous means ± shaded SEMs aggregated from n=3 biological replicates per condition.

(B-C) Representative western blot (panel B) and quantification (panel C) of VCAM-1 expression in BMEC-like cells cocultured with CRISPRi-astrocytes. CRISPRi-astrocytes were transduced with non-targeting sgRNA or dual sgRNAs targeting C3/SERPINA3. Samples were isolated on day 14 after coculture. GAPDH was used as a loading control. Quantification of western blot is graphed in panel C as mean ± SEM from n=3 biological replicates.

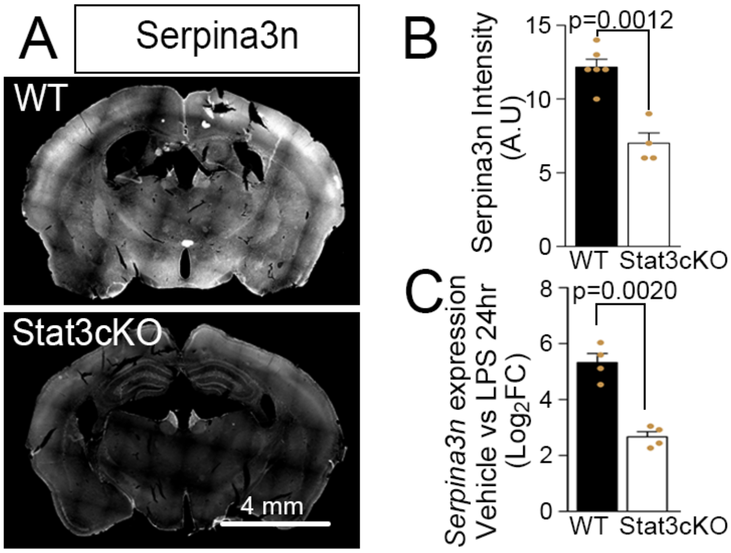

Supplementary Figure 7. Central nervous system expression of Serpina3n in wild-type versus Stat3cKO mice.

(A) Representative images of Serpina3n expression in wild-type (WT) versus mice lacking Stat3 in astrocytes (Stat3cKO).

(B) Quantification of Serpina3n signal intensity in brains of WT (n=6 mice) versus Stat3cKO (n=4 mice). Data are graphed as mean ± SEM. Statistical analyses were performed with a two-sided t-test.

(C) *Serpina3n* expression changes in astrocytes as a function of LPS treatment in WT versus Stat3cKO mice. Data were obtained from open access archival data from a previously published study[2]. Mice were given a peripheral dose of LPS or vehicle control and sacrificed after 24 hours. Astrocytes were then collected from spinal cord and subjected to bulk RNA sequencing. Data are presented as log_2FC between LPS and vehicle control for each condition. Data are graphed as mean ± SEM from n=4 mice. Statistical analyses were performed with a two-sided t-test.

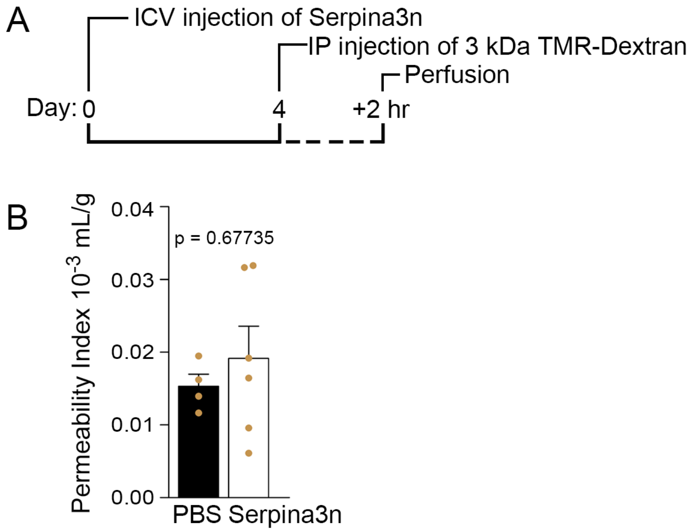

Supplementary Figure 8. 3 kDa dextran extravasation into brain after ICV injection of Serpina3n.

(A) Experimental description and timeline.

(B) Permeability index for mice receiving ICV injection of Serpina3n (n=6) versus PBS (n=4). Each data point represents an individual mouse, and data are presented as mean ± SEM. Statistical significance was calculated using an independent samples t-test.

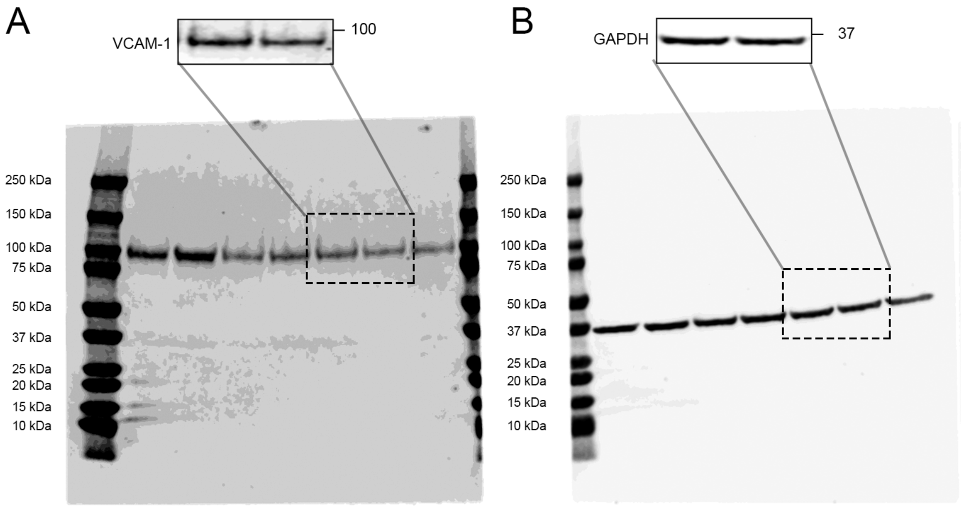

Supplementary Figure 9. Uncropped western blot for Supplementary Figure 5.

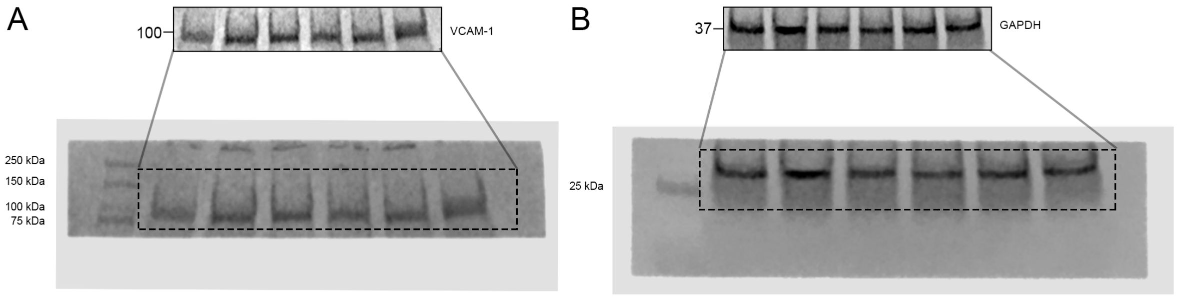

Supplementary Figure 10. Uncropped western blot for Supplementary Figure 6.

Supplementary References

1. Kang, B.H., Jensen, K.J., Hatch, J.A. & Janes, K.A. Simultaneous profiling of 194 distinct receptor transcripts in human cells. *Sci Signal* **6**, rs13 (2013).
2. Burda, J.E., O'Shea, T.M., Ao, Y., Suresh, K.V., Wang, S., Berstein, A.M., Chandra, A., Deverasetty, S., Kawaguchi, R., Kim, J.H., McCallum, S., Rogers, A., Wahane, S., & Sofroniew, M.V. Divergent transcriptional regulation of astrocyte reactivity across disorders. *Nature* **606**, 557-564 (2022).
