# Supplementary material for: Reactive astrocytes transduce inflammation in a blood-brain barrier model through a TNF-STAT3 signaling axis and secretion of alpha 1-antichymotrypsin
Source: Nat Commun. 2022 Nov 2;13:6581. doi: 10.1038/s41467-022-34412-4 (PMC9630454; doi:10.1038/s41467-022-34412-4)
Supplement: Supplementary file 2 — Reporting Summary [file 41467_2022_34412_MOESM2_ESM.pdf]

nature portfolio

Corresponding author(s): Ethan S. Lippmann

Last updated by author(s): Oct 21, 2022

Reporting Summary

Nature Portfolio wishes to improve the reproducibility of the work that we publish. This form provides structure for consistency and transparency in reporting. For further information on Nature Portfolio policies, see our Editorial Policies and the Editorial Policy Checklist.

Statistics

For all statistical analyses, confirm that the following items are present in the figure legend, table legend, main text, or Methods section.

n/a	Confirmed	
☐	☒	The exact sample size (*n*) for each experimental group/condition, given as a discrete number and unit of measurement
☐	☒	A statement on whether measurements were taken from distinct samples or whether the same sample was measured repeatedly
☐	☒	The statistical test(s) used AND whether they are one- or two-sided<br>*Only common tests should be described solely by name; describe more complex techniques in the Methods section.*
☒	☐	A description of all covariates tested
☐	☒	A description of any assumptions or corrections, such as tests of normality and adjustment for multiple comparisons
☐	☒	A full description of the statistical parameters including central tendency (e.g. means) or other basic estimates (e.g. regression coefficient) AND variation (e.g. standard deviation) or associated estimates of uncertainty (e.g. confidence intervals)
☐	☒	For null hypothesis testing, the test statistic (e.g. *F*, *t*, *r*) with confidence intervals, effect sizes, degrees of freedom and *P* value noted<br>*Give P values as exact values whenever suitable.*
☒	☐	For Bayesian analysis, information on the choice of priors and Markov chain Monte Carlo settings
☒	☐	For hierarchical and complex designs, identification of the appropriate level for tests and full reporting of outcomes
☐	☒	Estimates of effect sizes (e.g. Cohen's *d*, Pearson's *r*), indicating how they were calculated

Our web collection on statistics for biologists contains articles on many of the points above.

Software and code

Policy information about availability of computer code

Data collection	No software was used to collect data.
Data analysis	The following software is used to analyze the collected data.<br>Python v3.7<br>R V3.6<br>Rstudio V3.6.1<br>ggplot2 R package v3.3.0<br>Mapping by HISAT2 v.2.2.0<br>EdgeR v3.30.3<br>Limma v3.9<br>WEB-based Gene SeT AnaLasis Toolkit (WebGestalt) v2019<br>Fiji (ImageJ) v1.53f51, v1.53c<br>StarDist<br>STRING database v11.0b<br>Origene 2021b<br>GraphPad Prism v9

For manuscripts utilizing custom algorithms or software that are central to the research but not yet described in published literature, software must be made available to editors and reviewers. We strongly encourage code deposition in a community repository (e.g. GitHub). See the Nature Portfolio guidelines for submitting code & software for further information.

Data

Policy information about availability of data

All manuscripts must include a data availability statement. This statement should provide the following information, where applicable:

- Accession codes, unique identifiers, or web links for publicly available datasets
- A description of any restrictions on data availability
- For clinical datasets or third party data, please ensure that the statement adheres to our policy

RNA sequencing data have been uploaded to ArrayExpress under the accession number E-MTAB-11468 (https://www.ebi.ac.uk/biostudies/arrayexpress/studies/E-MTAB-11468). The human genome (hg19, Feb. 2009 GRCh37) is available from the NCBI Dataset Genome page.

Field-specific reporting

Please select the one below that is the best fit for your research. If you are not sure, read the appropriate sections before making your selection.

☒ Life sciences ☐ Behavioural & social sciences ☐ Ecological, evolutionary & environmental sciences

For a reference copy of the document with all sections, see nature.com/documents/nr-reporting-summary-flat.pdf

Life sciences study design

All studies must disclose on these points even when the disclosure is negative.

Sample size	(1) Human brain tissue<br>One unspecified non-surgical convulsive seizure case<br>One cerebral amyloid angiopathy case<br>Four Alzheimer's disease cases<br>Four age-matched asymptomatic non-demented cases<br>No statistical methods were used to predetermine sample sizes. The number of samples was determined based on the availability of human brain tissue.<br><br>(2) Serpina3n delivery into mouse brain via intracerebroventricular injection<br>Seventeen male mice randomly assigned into two arms for histology: eight treated with PBS and nine with Serpina3n.<br>Ten male mice randomly assigned into two arms for permeability assay: four treated with PBS and six with Serpina3n.<br>No statistical methods were used to predetermine sample sizes. Sample sizes were based on prior work using n=4-14 mice per group for studies of BBB inflammation and permeability (https://doi.org/10.1172/JCI140966).<br><br>(3) Evaluation of astrocyte reactivity and BBB integrity by immunostaining in Stat3-cKO mice<br>6 control and 4 Stat3-cKO mice (both male and female) were used.<br>No statistical methods were used to predetermine sample sizes. The number of samples was determined based on the availability of tissue.<br><br>(4) In vitro / ex vivo experiments<br>No statistical methods were used to predetermine sample sizes. All experiments were repeated at least three times to establish biological reproducibility similar to prior work (https://doi.org/10.1016/j.stemcr.2019.05.008).
Data exclusions	No acquired data was excluded from the statistical analyses.
Replication	All experiments using human or mouse tissue utilized at least 3 independent samples. All experiments performed in mice utilized at least 4 animals per group. Outcomes from all in vitro and ex vivo experiments were validated across at least 3 biological replicates.
Randomization	Animals were randomly assigned to experimental groups. In vitro/ex vivo samples were randomly segregated into experimental groups.
Blinding	Investigators were not blinded to group allocation during data collection and analyses because the experiments were not evaluated using subjective metrics and therefore not at risk for bias. For image analyses, where bias could potentially be an issue, automated macro scripts were used.

Reporting for specific materials, systems and methods

We require information from authors about some types of materials, experimental systems and methods used in many studies. Here, indicate whether each material, system or method listed is relevant to your study. If you are not sure if a list item applies to your research, read the appropriate section before selecting a response.

Materials & experimental systems

n/a	Involved in the study
	☒ Antibodies
	☒ Eukaryotic cell lines
☒	Palaeontology and archaeology
	☒ Animals and other organisms
	☒ Human research participants
☒	Clinical data
☒	Dual use research of concern

Methods

n/a	Involved in the study
☒	ChIP-seq
☒	Flow cytometry
☒	MRI-based neuroimaging

Antibodies

Antibodies used

Antibody details, dilution, Catalogue number, Vendor, Identifier, Clone number:
mouse anti-C3 (1:200 dilution, 846302, Biolegend, RRID:AB_2572128, 1H8/C3b)
rabbit anti-CD31 (1:200 dilution, RB-10333, Thermo, RRID:AB_720502)
mouse anti-CD44 (1:1000 dilution, AB6124, Abcam, RRID:AB_305297, F10-44-2)
mouse anti-Claudin-5 (1:1000 dilution, 352588, Invitrogen, RRID:AB_2532189, 4C3C2)
mouse anti-GBP2 (1:100 dilution, LS-B12172-50, LSBio, 5C8)
rabbit anti-GFAP (1:300 dilution, Z0334, Dako, RRID:AB_10013382)
mouse anti-GFAP (1:300 dilution, MAB360, Millipore, RRID:AB_11212597, GA5)
chicken anti-GFAP (1:300 dilution, SKU: GFAP, Aves, RRID:AB_2313547, N206A/8)
goat anti-GFP (1:1000 dilution, 600-141-215, Rockland, AB_1961516)
mouse anti-Glut1 (1:1000 dilution, FAB1418G, R&D Systems, 202915)
mouse anti-occludin (1:500 dilution, 33-1500, Thermo Fisher Scientific, RRID:AB_2533101, OC-3F10)
rabbit anti-pSTAT3(y705) (1:300 dilution, 9145S, Cell Signaling, RRID:AB_2491009, D3A7)
goat anti-Serpina3n (1:200 dilution, AF4709-SP, R&D Systems)
rabbit anti-VCAM-1 (1:200 dilution, ab134047, Abcam, RRID:AB_2721053, EPR5047)
mouse anti-VCAM-1 (1:200 dilution, sc-13160, Santa Cruz, RRID:AB_626846, E-10)
goat anti-VE-cadherin (1:300 dilution, AF938, R&D Systems, RRID:AB_355726)
sheep anti-E-selectin (1:1000 dilution, AF724, R&D Systems, RRID:AB_355549)
rabbit anti-GAPDH (1:5000 dilution, 5174, Cell signaling, D16H11, RRID:AB_10622025)
Goat anti-Mouse IgG, Alexa Fluor488 (1:1000 dilution, Thermo, A-11001, RRID:AB_2534069)
Goat anti-Rabbit IgG, Alexa Fluor488 (1:1000 dilution, Thermo, A-11008, RRID:AB_143165)
Goat anti-Chicken IgY, Alexa Fluor488 (1:1000 dilution, Thermo, A-11039, RRID:AB_2534096)
Goat anti-Rabbit IgG, Alexa Fluor546 (1:1000 dilution, Thermo, A-11035, RRID:AB_2534093)
Goat anti-Mouse IgG, Alexa Fluor546 (1:1000 dilution, Thermo, A-11030, RRID:AB_2534089)
Goat anti-Mouse IgG, Alexa Fluor647 (1:1000 dilution, Thermo, A32728, RRID:AB_2633277)
Goat anti-Rabbit IgG, Alexa Fluor647 (1:1000 dilution, Thermo, A32733, RRID:AB_2633282)
Goat anti-Chicken IgY, Alexa Fluor647 (1:1000 dilution, Thermo, A32933, RRID:AB_2762845)

Validation

To select primary antibodies, we used BenchSci to determine if the product had been used in published studies and evaluated the application to see if the antibody would be appropriate for use in our study. If the antibody had not been used in a prior publication, we relied on a validation statement provided on the manufacturer's website and then evaluated expected antibody performance.

mouse anti-C3: validation statement provided on manufacturer's website and expected staining patterns observed in human tissue
rabbit anti-CD31: used in 6 publications (BenchSci)
mouse anti-CD44: used in 48 publications (BenchSci)
mouse anti-claudin-5: used in 186 publications (BenchSci)
mouse anti-GBP2: validation statement provided on manufacturer's website and expected staining patterns were observed in iPSC-derived astrocytes
rabbit anti-GFAP: used in >2,300 publications (BenchSci)
mouse anti-GFAP: used in 766 publications (BenchSci)
chicken anti-GFAP: used in 81 publications (BenchSci)
goat anti-GFP: used in 19 publications (manufacturer's website)
mouse anti-Glut1: used in 1 publication (BenchSci) and and expected staining patterns observed in mouse tissue
mouse anti-occludin: used in 284 publications (BenchSci)
rabbit anti-pSTAT3: used in 1,200 publications (BenchSci)
goat anti-Serpina3n: used in 11 publications (BenchSci)
rabbit anti-VCAM-1: used in 186 publications (BenchSci)
mouse anti-VCAM-1: used in 57 publications (BenchSci)
goat anti-VE-cadherin: used in 23 publications (BenchSci)
sheep anti-E-selectin: used in 1 publication (BenchSci)
rabbit anti-GAPDH: used in 1,300 publications (BenchSci)

Eukaryotic cell lines

Policy information about cell lines

Cell line source(s)	CC3 iPSCs, WTC11 iPSCs, HEK293T
Authentication	iPSC lines were confirmed as karyotypically normal before cryopreservation of stock vials. Differentiated progeny were authenticated through immunostaining of expected markers and functional assays. HEK293T cells (used solely for virus production) were not authenticated.
Mycoplasma contamination	iPSC and HEK293T cell lines were not routinely tested for mycoplasma contamination.
Commonly misidentified lines (See ICLAC register)	HEK293T

Animals and other organisms

Policy information about studies involving animals; ARRIVE guidelines recommended for reporting animal research

Laboratory animals	1) Mice, wild-type C57BL/6J, male, 16-18 weeks old<br>2) Mice, Stat3cKO, male and female, 2-4 months old<br>3) Mice, wild-type C57BL/6J, male and female, 6 days old<br><br>Mice were housed in a controlled environment (68-76 degrees F, 30-70% humidity) on 12 hour light/dark cycles (lights on at 7am) and provided with regular chow and sterilized tap water ad libitum.
Wild animals	No wild animals were used in this study.
Field-collected samples	No field collected samples were used in the study.
Ethics oversight	All procedures were approved by the Vanderbilt IACUC or the UCLA IACUC.

Note that full information on the approval of the study protocol must also be provided in the manuscript.

Human research participants

Policy information about studies involving human research participants

Population characteristics	For human tissue samples, only post-mortem samples were used.<br>(1) One unspecified non-surgical convulsive seizure case. De-identified brain tissue from a patient with unspecified non-surgical convulsive seizures was obtained via Vanderbilt University Medical Center Cooperative Human Tissue Network.<br>(2) One cerebral amyloid angiopathy case (man;62 years) and four Alzheimer's disease cases and four age-matched asymptomatic non-demented cases (five women, three men; age range 62-81 years). Brain samples were collected with written consent for brain donation obtained from patients or their surrogate decision-makers. Brain tissue collection procedures were approved by the Institutional Review Board at Vanderbilt University Medical Center.
Recruitment	No recruitment strategies were used.
Ethics oversight	All tissue collection was approved by the Institutional Review Board at Vanderbilt University Medical Center.

Note that full information on the approval of the study protocol must also be provided in the manuscript.
