# Supplementary material for: Reactive astrocytes transduce inflammation in a blood-brain barrier model through a TNF-STAT3 signaling axis and secretion of alpha 1-antichymotrypsin
Source: Nat Commun. 2022 Nov 2;13:6581. doi: 10.1038/s41467-022-34412-4 (PMC9630454; doi:10.1038/s41467-022-34412-4)
Supplement: Supplementary file 3 — Source Data [file 41467_2022_34412_MOESM3_ESM.zip › Source data - uncropped western blots from Figure 4.pdf]

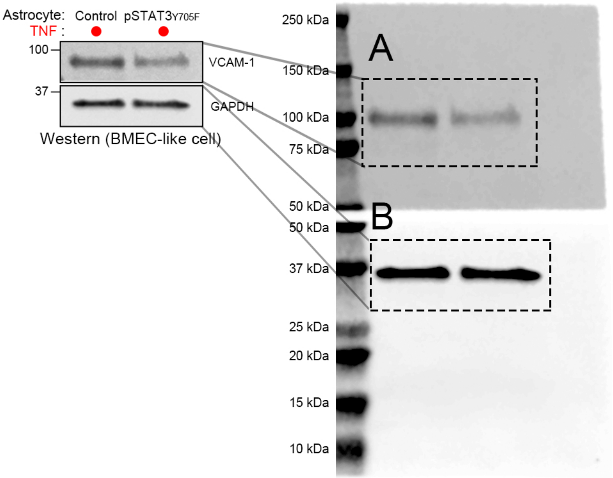
Astrocyte: Control pSTAT3Y705F
TNF :
100
37
VCAM-1
GAPDH
Western (BMEC-like cell)
250 kDa
150 kDa
100 kDa
75 kDa
50 kDa
50 kDa
37 kDa
25 kDa
20 kDa
15 kDa
10 kDa
A
B
