# Supplementary figures and images for: Reactive astrocytes transduce inflammation in a blood-brain barrier model through a TNF-STAT3 signaling axis and secretion of alpha 1-antichymotrypsin
Source: Nat Commun. 2022 Nov 2;13:6581. doi: 10.1038/s41467-022-34412-4 (PMC9630454; doi:10.1038/s41467-022-34412-4)

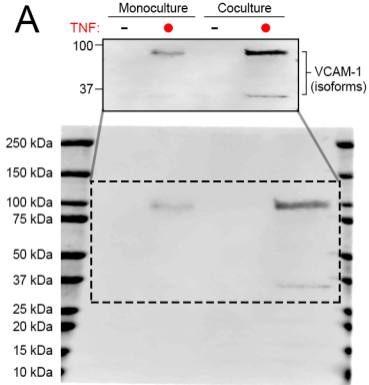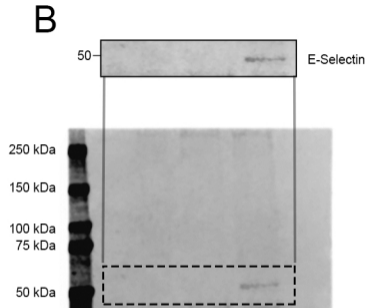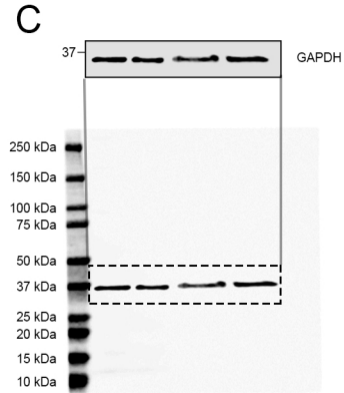

Supplement: Supplementary file 3 — Source Data [file 41467_2022_34412_MOESM3_ESM.zip › Source data - uncropped western blots from Figure 3.pdf]

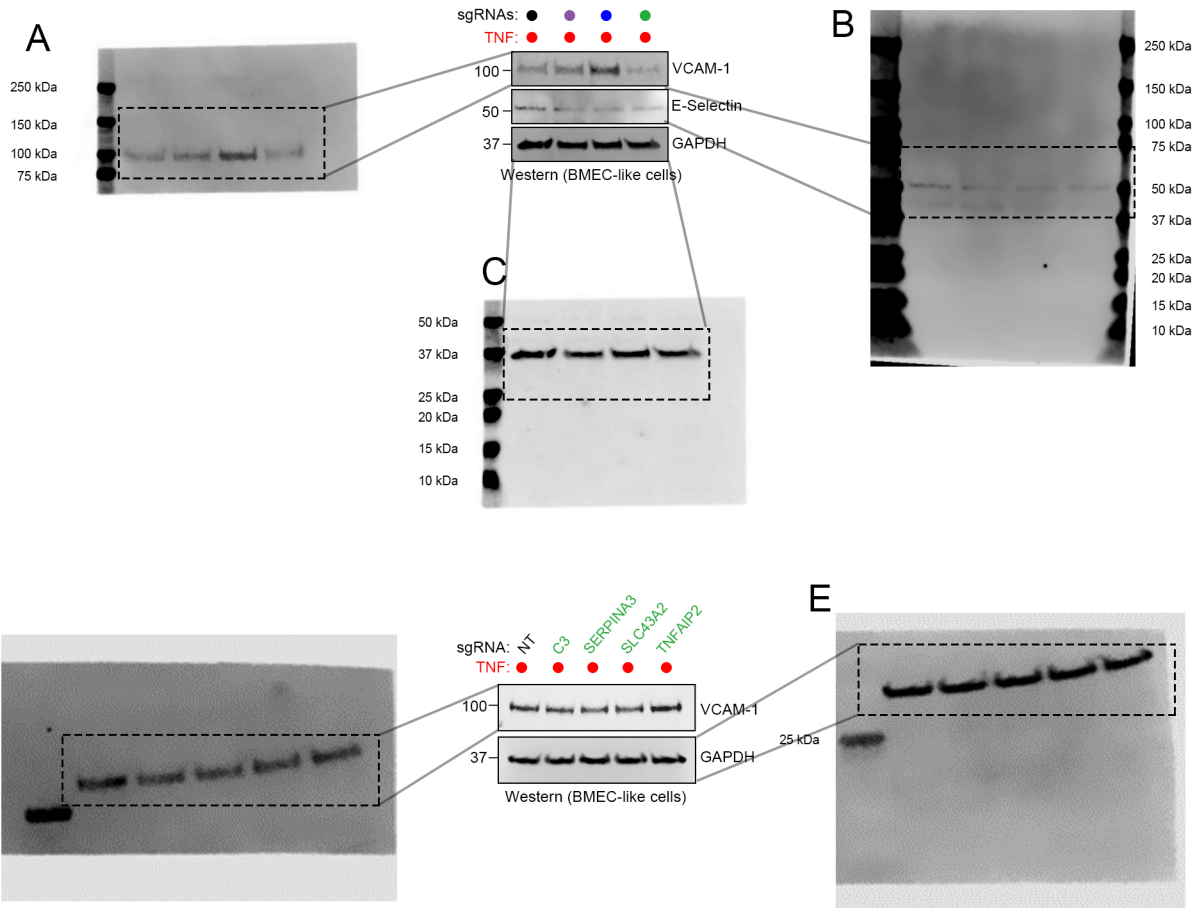

Supplement: Supplementary file 3 — Source Data [file 41467_2022_34412_MOESM3_ESM.zip › Source data - uncropped western blots from Figure 5.pdf]
